# MetMaxStruct: A Tversky-Similarity-Based Strategy for Analysing the (Sub)Structural Similarities of Drugs and Endogenous Metabolites

**DOI:** 10.3389/fphar.2016.00266

**Published:** 2016-08-22

**Authors:** Steve O'Hagan, Douglas B. Kell

**Affiliations:** ^1^School of Chemistry, The University of ManchesterManchester, UK; ^2^The Manchester Institute of Biotechnology, The University of ManchesterManchester, UK; ^3^Manchester Centre for Synthetic Biology of Fine and Speciality Chemicals, The University of ManchesterManchester, UK

**Keywords:** drug transporters, cheminformatics, Tversky similarity, endogenites, metabolomics

## Abstract

**Background:** Previous studies compared the molecular similarity of marketed drugs and endogenous human metabolites (endogenites), using a series of fingerprint-type encodings, variously ranked and clustered using the Tanimoto (Jaccard) similarity coefficient (TS). Because this gives equal weight to all parts of the encoding (thence to different substructures in the molecule) it may not be optimal, since in many cases not all parts of the molecule will bind to their macromolecular targets. Unsupervised methods cannot alone uncover this. We here explore the kinds of differences that may be observed when the TS is replaced—in a manner more equivalent to semi-supervised learning—by variants of the asymmetric Tversky (TV) similarity, that includes α and β parameters.

**Results:** Dramatic differences are observed in (i) the drug-endogenite similarity heatmaps, (ii) the cumulative “greatest similarity” curves, and (iii) the fraction of drugs with a Tversky similarity to a metabolite exceeding a given value when the Tversky α and β parameters are varied from their Tanimoto values. The same is true when the sum of the α and β parameters is varied. A clear trend toward increased endogenite-likeness of marketed drugs is observed when α or β adopt values nearer the extremes of their range, and when their sum is smaller. The kinds of molecules exhibiting the greatest similarity to two interrogating drug molecules (chlorpromazine and clozapine) also vary in both nature and the values of their similarity as α and β are varied. The same is true for the converse, when drugs are interrogated with an endogenite. The fraction of drugs with a Tversky similarity to a molecule in a library exceeding a given value depends on the contents of that library, and α and β may be “tuned” accordingly, in a semi-supervised manner. At some values of α and β drug discovery library candidates or natural products can “look” much more like (i.e., have a numerical similarity much closer to) drugs than do even endogenites.

**Conclusions:** Overall, the Tversky similarity metrics provide a more useful range of examples of molecular similarity than does the simpler Tanimoto similarity, and help to draw attention to molecular similarities that would not be recognized if Tanimoto alone were used. Hence, the Tversky similarity metrics are likely to be of significant value in many general problems in cheminformatics.

## Introduction

It is widely recognized that drugs exploit or “hitchhike on” protein transporters in order to be taken up into cells (e.g., Ecker and Chiba, 2009; Giacomini et al., 2010; Fromm and Kim, 2011; Giacomini and Huang, 2013; Ishikawa et al., 2013; Sugiyama and Steffansen, 2013; Ecker, 2014; You and Morris, 2014). However, it is not at all easy to predict which transporters are used simply by looking at the chemical structures of the drugs. As part of a series of studies of the transporter-mediated uptake of pharmaceutical drugs into biological cells (e.g., Dobson and Kell, 2008; Dobson P. et al., 2009; Kell and Dobson, 2009; Kell et al., 2011, 2013, 2015; Lanthaler et al., 2011; Kell, 2013, 2015a,b, 2016a,b; Kell and Goodacre, 2014; Mendes et al., 2015; Kell and Oliver, 2014; O'Hagan and Kell, 2015a), and driven by the availability of principled metabolic network reconstructions (Herrgård et al., 2008; Swainston et al., 2013; Thiele et al., 2013; Sahoo et al., 2014; Nigam, 2015; Palsson, 2015) (in which approximately one third of the enzymes are transporters), we have been developing the consequent idea that drugs do indeed share structural similarities with endogenous metabolites (“endogenites”; Dobson P. D. et al., 2009; O'Hagan and Kell, 2015c; O'Hagan et al., 2015). The implication would be that the natural (endogenite) substrates are those with which the drugs share the more significant molecular similarities. These latter studies, comparing drug-endogenite structures were purely “unsupervised,” and thus based on clustering-type comparisons. This was because (i) we wished to avoid any dangers of overtraining using a supervised method, and (ii) in relatively few cases do we in fact know the natural (endogeneous) substrates of those “SLC” (SoLute Carrier) transporters (Hediger et al., 2013; César-Razquin et al., 2015) that can be shown to transport drug molecules. A recent example of this latter is SLC35F2, that is responsible for rather more than 99% of the transport of the anti-cancer drug candidate YM155 (Winter et al., 2014), but whose endogenous substrate is unknown. In a related vein, it has been argued (with evidence) that the “natural” substrate of the OCTN1/SLC22A4 transporter (Koepsell, 2013) is not (as was widely believed) carnitine but instead the dietary and/or microbial product ergothioneine (Gründemann et al., 2005; Gründemann, 2012).

In some cases the structural similarities between drugs and endogenites are sufficiently close that it is clear which transporters are the most likely candidates, but this is not always the case. Although empirical (experimental) methods are coming forward that can help us find the relevant transporters more or less systematically (e.g., Lanthaler et al., 2011; Winter et al., 2014; César-Razquin et al., 2015), mostly we lack the means to generate good hypotheses for which transporters transport which drugs. The basic problem is that the *purely* unsupervised structural comparisons using Tanimoto similarities are based on *the whole molecule*, and substructures that are irrelevant (or not directly bound to the transporter protein when being transported) serve to act as skillful decoys. Specifically, and rather obviously, in the cases of proteins binding small molecules, any part of the small molecule that does not actually bind to the protein is unlikely to contribute much to its biological activity.

Supervised methods—that in cheminformatics amount to Quantitative Structure-Activity Relationships (QSARs; Sedykh et al., 2013; Cherkasov et al., 2014; Ruusmann et al., 2014)—are much more powerful than are unsupervised methods, but can hardly be applied when we do not know the relevant substrates nor (thus) have any assay data. However, besides strictly unsupervised and supervised learning, there is a third class of computational analysis, known as semi-supervised learning (e.g., Demiriz et al., 1999; Handl and Knowles, 2006; Zhu and Goldberg, 2009; Balcan and Blum, 2010; Chapelle et al., 2010; Kingma et al., 2014), in which one uses a surrogate objective function for unlabeled data where they are available, even when one does not know the true class membership (here, for instance substrate or inhibitor activity) that one is actually seeking in order to improve one's understanding of a system. Here, we recognize that the “surrogate” objective function may simply be a greater (or different) similarity coefficient when something is varied. Although not necessarily new in this context (Broomhead and Lowe, 1988; Moody and Darken, 1989), these “mixed” strategies have recently come to the fore in cases (e.g., Hinton and Salakhutdinov, 2006; Hinton et al., 2006; Hinton, 2007) where one uses an unsupervised method as (a preparatory) part of the training of a supervised system, in particular a deep neural learning system (Bengio, 2009; Erhan et al., 2010; Lecun et al., 2015).

A similar question relates to the choice of which kinds of molecules one might use in an experimental QSAR study given an initial hit or lead, and one answer must include molecules that bear at least some structural similarities to the initial hit/lead. Again, just basing the choice on an overall similarity is likely to mean that some molecules that contain a similar scaffold may appear to have a TS that is quite different from that of the initial hit and thus are not chosen. We clearly need “better” and more general methods for assessing “similarity,” where we recognize that the concept of “better” implies an objective function (and we give an example below).

As mentioned, the inevitable flaw in *purely* unsupervised methods is that they (can) have no knowledge of which parts of an input (e.g., substructures of a molecular structure) are “important” to (or correlate with) an output (process) of interest and which parts are not, because that is not the question being asked (Broadhurst and Kell, 2006; Hastie et al., 2009). The equivalent comparison in linear multivariate statistics is between principal components analysis (unsupervised) and partial least squares analysis (supervised; Wold et al., 2001). For the former, various kinds of normalization can be used to upweight or downweight particular features (e.g., Hotelling, 1933; Neal et al., 1994). This issue is particularly acute in standard cheminformatics, where the Tanimoto (Jaccard) coefficient is commonly used as an index of molecular similarity following fingerprints encoding, and where the numerical similarity returned is dominated by the number of bits set to 1 in the output comparator string (and hence is also a reflection of molecular size; Flower, 1998; Willett et al., 1998; Dixon and Koehler, 1999; Salim et al., 2003; Willett, 2006; Wang et al., 2007; Wang and Bajorath, 2008; Senger, 2009; O'Hagan and Kell, 2015c). In the case of drug-endogenite similarity measurements, this can often tend to favor particular endogenites that happen to share many chemical groupings with the drugs of interest; CoA derivatives fall (and fell O'Hagan et al., 2015) into this category, at least for certain cheminformatics encodings. We note, as pointed out by a referee, that the MACSS encoding was originally devised for cataloging chemicals; this said, it has been widely used for providing a computer-readable encoding for both similarity searches and even QSARs.

**Figure 1:**
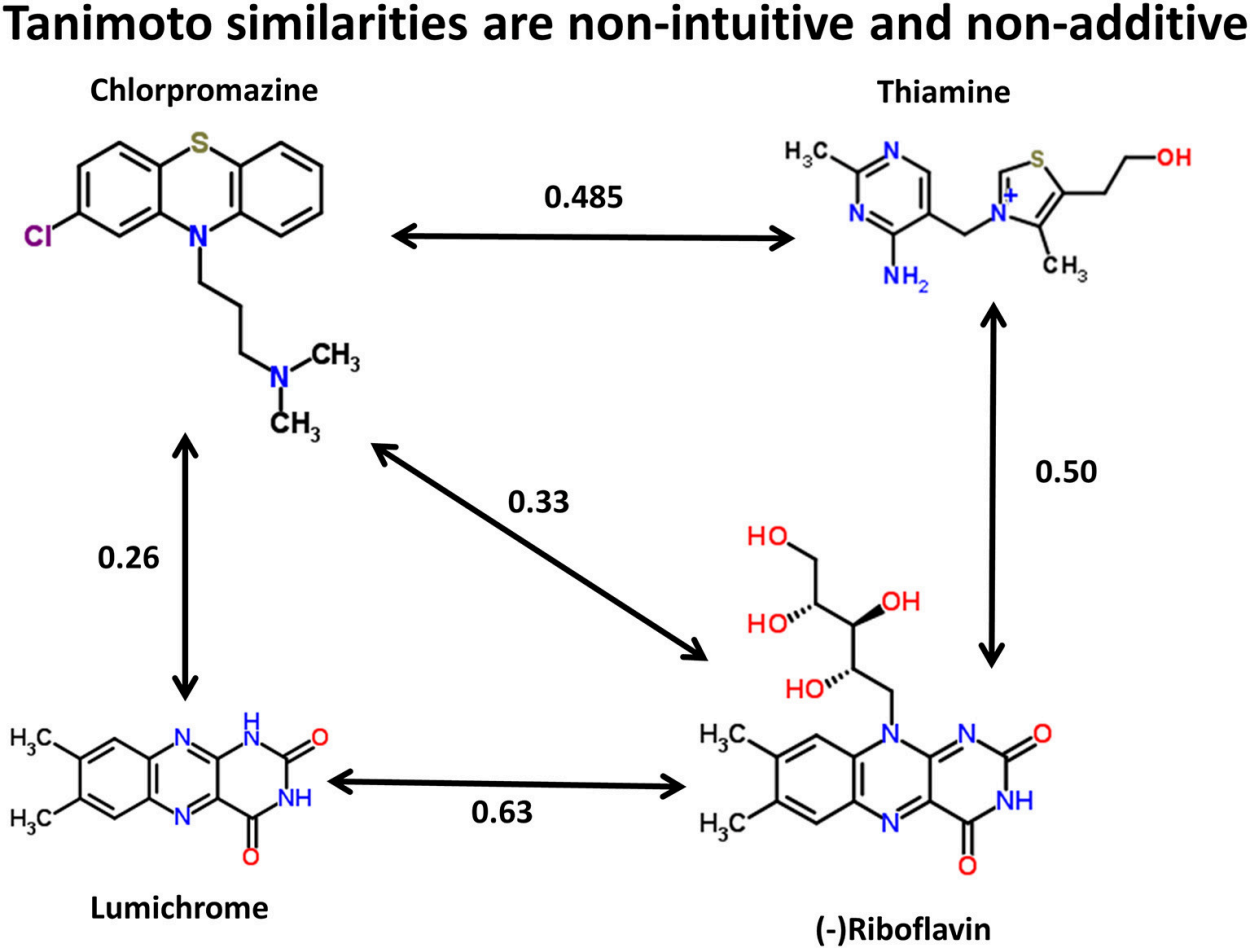
**Tanimoto similarities between chlorpromazine and three other molecules (using the MACCS166 encoding)**.

We can illustrate the basic principle (using the data available in the Supplementary Materials to (O'Hagan et al., 2015), and the kind of comparison illustrated for propranolol vs. endogenites in **Figure 3** of that paper) by three of the structures in Figure 1. Thus, using the MACCS166 encoding (Durant et al., 2002), and chlorpromazine as the interrogatory drug, the top endogenite returned is thiamine. However, visual inspection of the structure of riboflavin (vitamin B_2_), for instance, suggests that its tricyclic core is actually rather more similar to that of chlorpromazine (as has indeed occasionally been noted functionally Gabay and Harris, 1965; Pinto et al., 1981; Pelliccione et al., 1983; Tomei et al., 2001; Iwana et al., 2008; Caldinelli et al., 2010; Iwasa et al., 2011), but the Tanimoto similarity is both lower and potentially depressed by the ribitol sidechain. Nonetheless, removing the ribitol sidechain (to give lumichrome) actually lowers the Tanimoto similarity to chlorpromazine, consistent with the comments above regarding molecular size and Tanimoto similarity. In other words, (i) visual appearance can be a poor guide to calculated chemical similarity, (ii) one would here desire a method or methods that can pick up on a large change in a (small) part of a molecule that it otherwise still recognizes as being similar, and (iii) as pointed out by a referee the similarity coefficient necessarily depends on the encoding chosen (for reasons of space we use solely the MACCS166 encoding here).

Molecular similarity necessarily depends on context (Bender and Glen, 2004), and as we detailed earlier could differ quite widely for the same pairs of molecules as the encoding was varied. Given that our fundamental question (O'Hagan and Kell, 2015c; O'Hagan et al., 2015) is “which is the endogenite that is closest in molecular structure, in some sense, to a given drug molecule X?,” it is clear that what is needed is some kind of an automated analysis of this type. This would exploit information on selected parts of the molecule that might, when assessed “correctly,” be found to be more endogenite-like than when the assessment is made using the entire molecules. Thus, in general terms, it could look for substructures of drugs that *increase* the (Tanimoto or other) similarity of at least some metabolites relative to that based on their overall structure. These would thereby generate hypotheses that return those endogenous metabolites that are more likely (than the “overall most similar molecules” returned) to represent good suggestions for particular purposes, even if, during the computational analyses, we do not have measures of (i.e., the values for) those purposes. Holliday et al. (2002) provide a list of 22 similarity measures that have been used in cheminformatics, although they do not include the Tversky similarities on which we concentrate below.

The Tanimoto (Jaccard) similarity of a set of (typically binary) attributes is a true metric, defined as their intersection divided by their union, and is given (for simple bitstrings of the same length) as:

(1) $$\begin{array}{l} \frac{M_{11}}{M_{10}+M_{01}+M_{11}} \end{array}$$

where *M*_11_ is the number of positions in which both bits are set to 1 while the sum of *M*_10_ plus *M*_01_ together represent the number of positions in the reciprocal cases in which they are different.

Equivalently, if the number of bits set to 1 in A but to 0 in B is *a*, the number of those in B set to 1 but not in A is *b*, and those both set to 1 is *c*, the Tanimoto similarity TS between two bitstrings A and B is given by:

(2) $$\begin{array}{l} \text{TS}(\text{A},\text{B})=\frac{c}{a+b+c} \end{array}$$

Simple inspection indicates that the Tanimoto similarity ranges from 0 (complete lack of similarity) to 1 (identity). However, a more general method of similarity assessment is that due to Tversky (1977).

The Tversky similarity coefficient (Tversky, 1977; Senger, 2009; Geitmann et al., 2011; Gan et al., 2014; or, more accurately, sets of similarity coefficients) represent, in a sense, a more discriminating and asymmetric variant of the Tanimoto similarity in which we might not wish to make the comparison over the whole molecule. This is done by introducing additional parameters α and β. The Tversky similarity coefficient Tv(A,B) is then defined as:

(3) $$\begin{array}{l} \text{Tv}(\text{A},\text{B})=\text{c}/(\alpha \text{a}+\;\beta \text{b}+\text{c}) \end{array}$$

where again *a* and *b* are the number of bits that are set to be “on” (1 bits) only in molecular fingerprints A or B, respectively, and c is the number of on bits shared by both A and B. For these purposes, A is an interrogatory molecule while B is the molecule being interrogated as to its similarity. It is common, but not necessary, to vary α and β such that α + β = 1. The smaller the value of α, the larger the contribution of B as a substructure of A (and hence to its similarity with A). The larger the value of α, the larger the contribution of B as a superstructure of A (equivalently A as a substructure of B). For α = β = 1 the coefficient is numerically equivalent to the Tanimoto similarity, while the coefficient when α (= β) = 0.5 is known as the Dice coefficient. Clearly, then, and as a simple extension of our previous Tanimoto-based analyses (O'Hagan and Kell, 2015c; O'Hagan et al., 2015), it is likely to be worth studying the effects of substituting the Tanimoto coefficient by various values of the Tversky coefficient to understand which kinds of drug molecules may begin to appear more similar to endogenites when α ≠ 1. This is the purpose of the present paper.

We note that there have been comparatively few systematic studies of this general topic, and none at all comparing marketed drugs and endogenites. An extension of this is also precisely the motivation (Riniker and Landrum, 2013) behind the “fraggle” algorithm, for which we cannot find a published reference, but which is explained at https://github.com/rdkit/UGM_2013/blob/master/Presentations/Hussain.Fraggle.pdf. Here, our desire for “good suggestions” hinges on what are, in fact, the endogenous substrates of relevant transporters. It turns out that one can use this general strategy to improve the similarity to at least one endogenite for a great many marketed drugs. This obviously might have a substantial and useful effect on the endogeneous metabolites (or other molecules) one might seek to test for their role as substrates (or indeed inhibitors) of the drug transporter activity of specific proteins.

## Materials and methods

The list of endogenites derive from Recon2 (Thiele et al., 2013) and the full list of marketed drugs taken from DrugBank (Law et al., 2014) are those that were given previously (O'Hagan et al., 2015) and are all available in the Supplementary Materials to O'Hagan et al. (2015). In a similar vein, as before (O'Hagan and Kell, 2015b,c; O'Hagan et al., 2015), we used the KNIME software (see http://knime.org/ and e.g., Berthold et al., 2008; Mazanetz et al., 2012; Beisken et al., 2013) to create workflows for our analyses. In particular, substantial use was made of the RDKit nodes (see http://rdkit.org/ and e.g., Landrum et al., 2011; Landrum and Stiefl, 2012; Riniker and Landrum, 2013; Riniker et al., 2013, 2014; O'Hagan and Kell, 2015b), noting the very useful “fraggle” (http://www.rdkit.org/Python_Docs/rdkit.Chem.Fraggle-module.html). The Tv similarity calculations were obtained using a node from the Indigo library (see Saubern et al., 2011).

## Results

**Figure 2:**
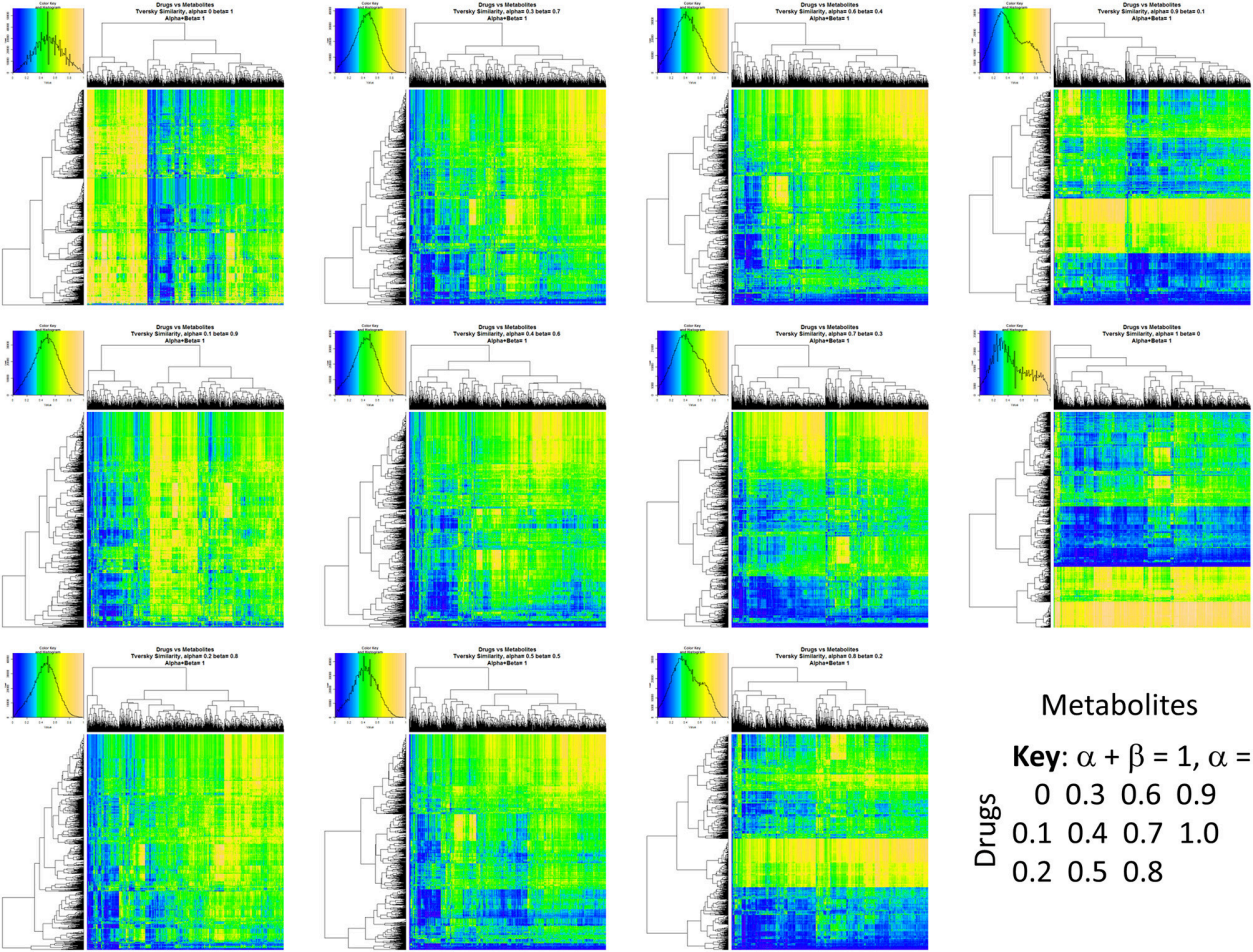
**Eleven heatmaps showing color-encoded drug-endogenite Tversky similarities for Tversky α + β = 1**. The figure is intended to give an easy overview, with similarities ranging from 0 in dark blue to 1 in buff orange as per the color map inserts. The key for the varying values of α is given in the lower right-hand corner of the figure.

**Figure 3:**
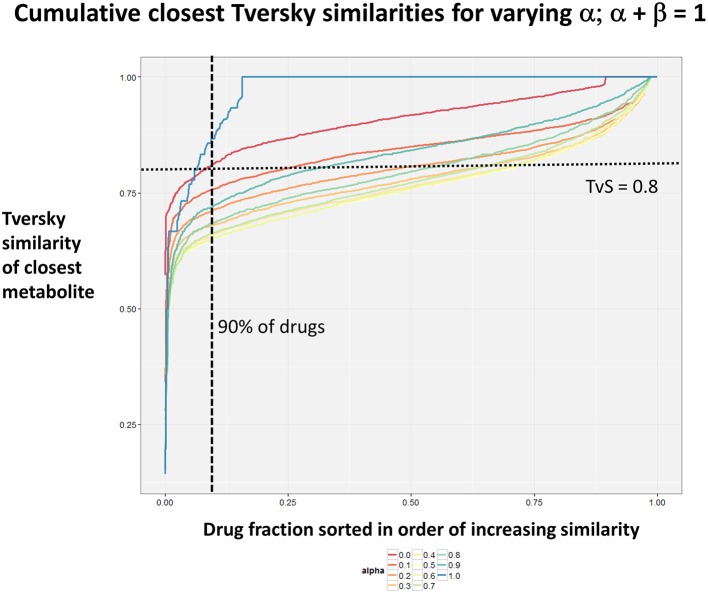
**Cumulative plot of drug-endogenite likenesses using varying values of the Tversky similarity coefficient α with the constraint α + β = 1**. For each curve, the maximum Tversky similarity to any metabolite for each drug is plotted in rank order, starting from the right. It is obvious that, especially for values of α closest to 0 or 1, there is an endogenite that is really very similar to the interrogating drug, and much more similar than those found (O'Hagan et al., 2015) when the metric is the Tanimoto similarity.

**Figure 4:**
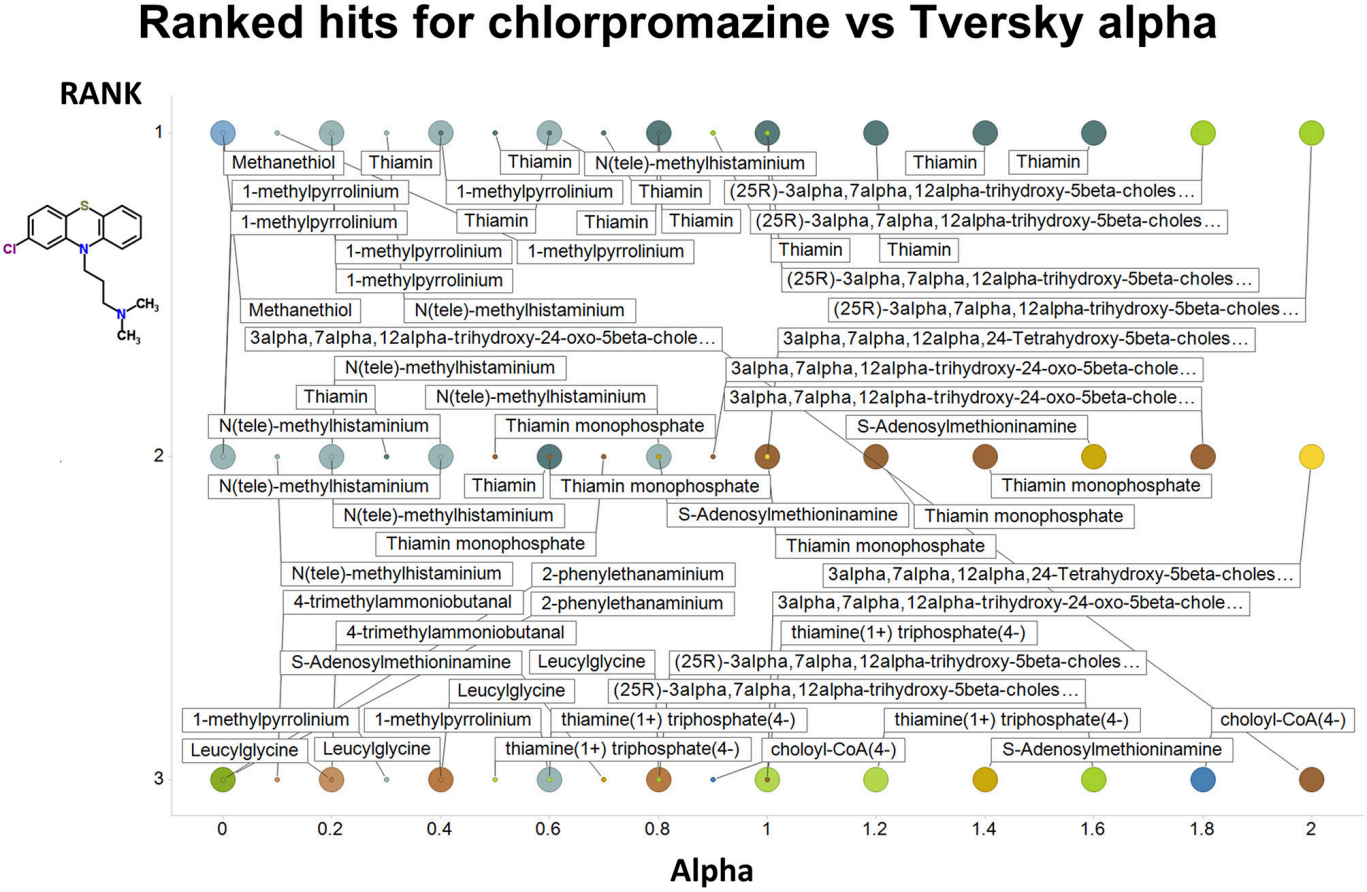
**Top 3 ranked hits for the closest endogenites to chlorpromazine at different values of the Tversky α for both α + β = 1 and α + β = 2**. Two example points are given for α = 0, 0.2, 0.4, 0.6 0.8, and 1.0; the smaller circle is for α + β = 1. It is clear (as expected) that the top-ranked hits become more complex as α increases.

**Figure 5:**
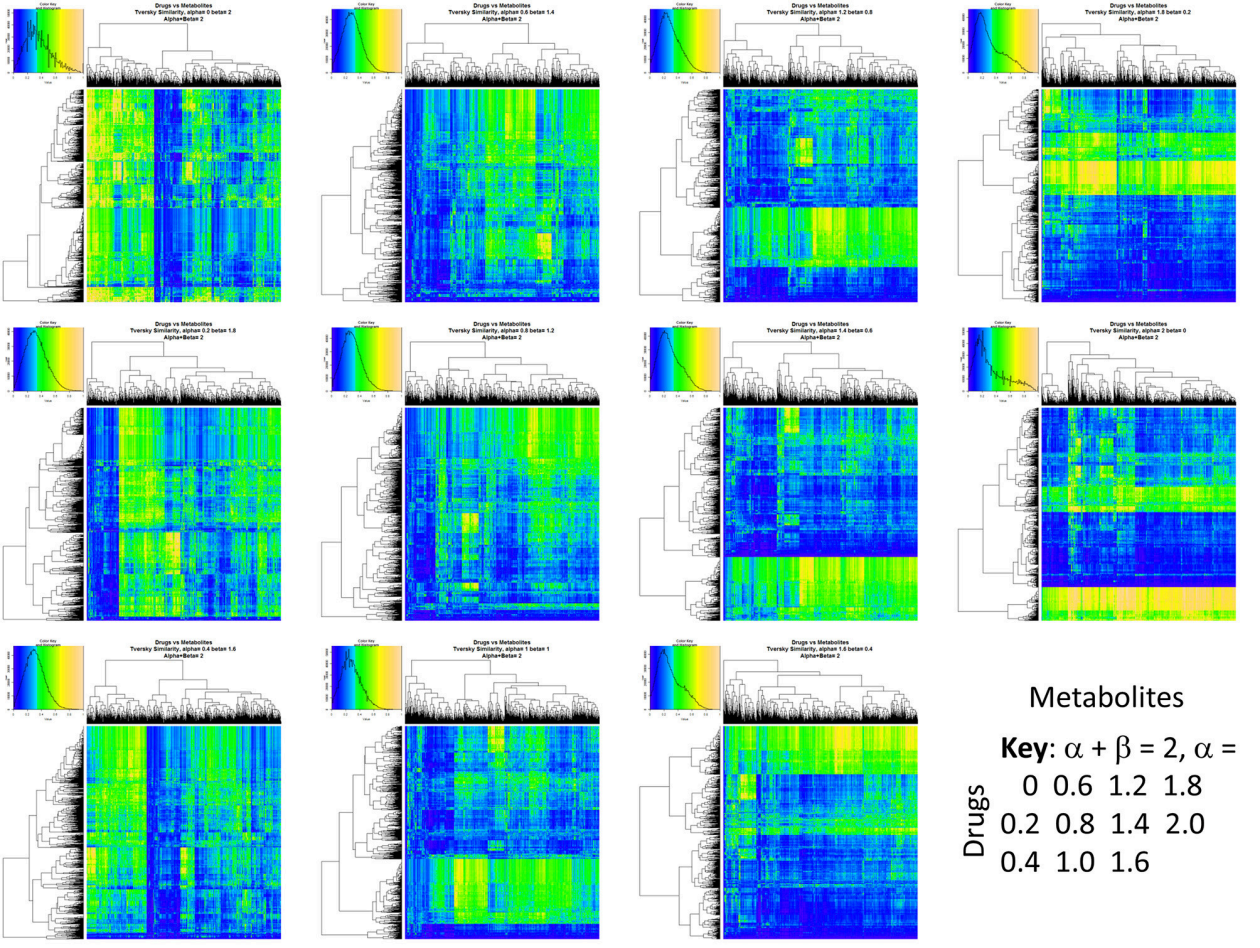
**Eleven heatmaps showing color-encoded drug-endogenite Tversky similarities for Tversky α + β = 2**. The figure is intended to give an easy overview, with similarities ranging from 0 in dark blue to 1 in buff orange as per the color map inserts. The key for the varying values of α is given in the lower right-hand corner of the figure. The basic experiment is otherwise exactly the same as that in Figure 2.

**Figure 6:**
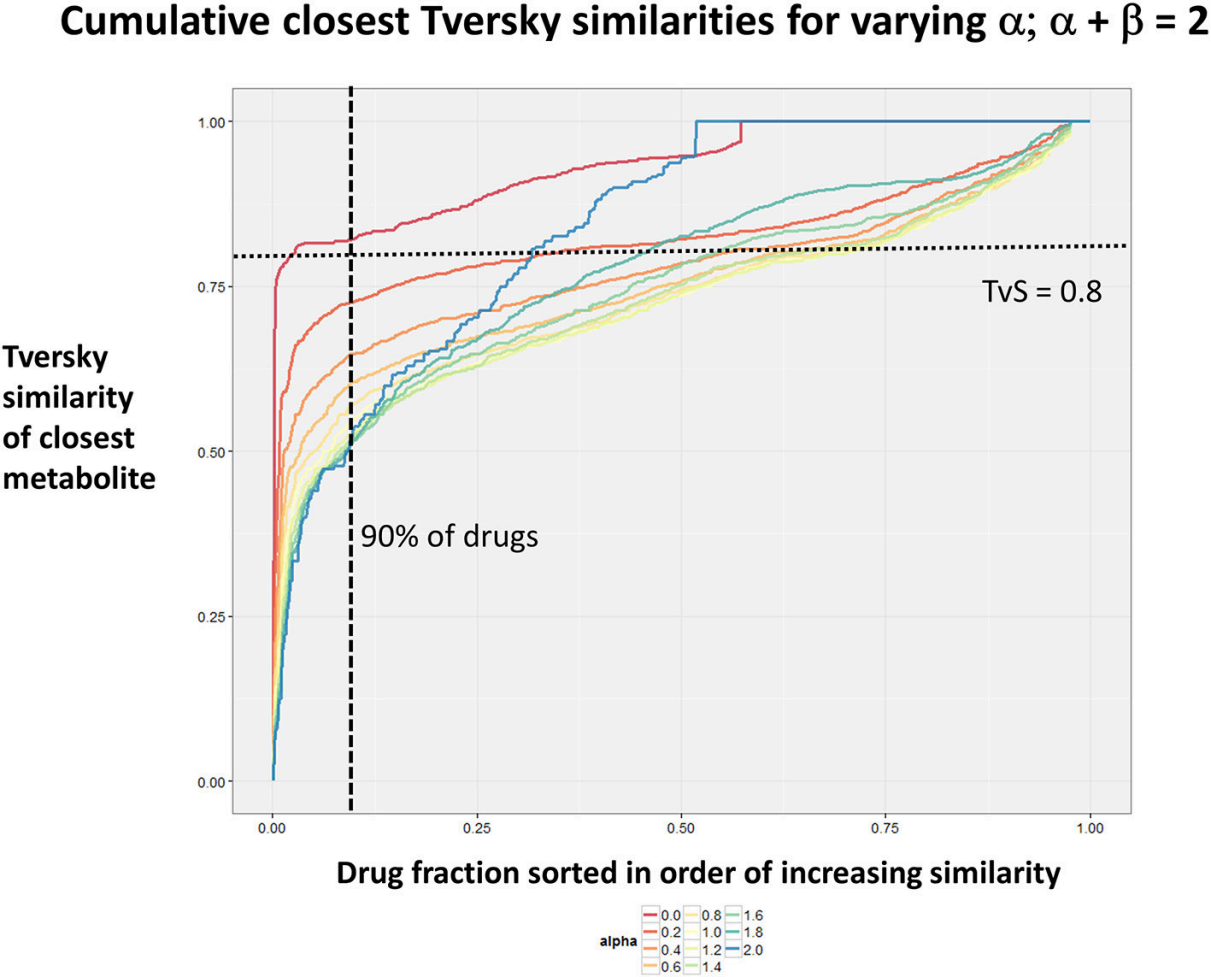
**Cumulative plot of drug-endogenite likenesses using varying values of the Tversky similarity coefficient for α + β = 2**. For each curve, the maximum Tversky similarity to any metabolite for each drug is plotted in rank order, starting from the right. It is obvious that, especially for values of α closest to 0 or 2, there is an endogenite that is really very similar to the interrogating drug, and more similar than those found (O'Hagan et al., 2015) when the metric is the Tanimoto similarity. However, the similarities are always greater when α + β = 1 (Figure 2).

**Figure 7:**
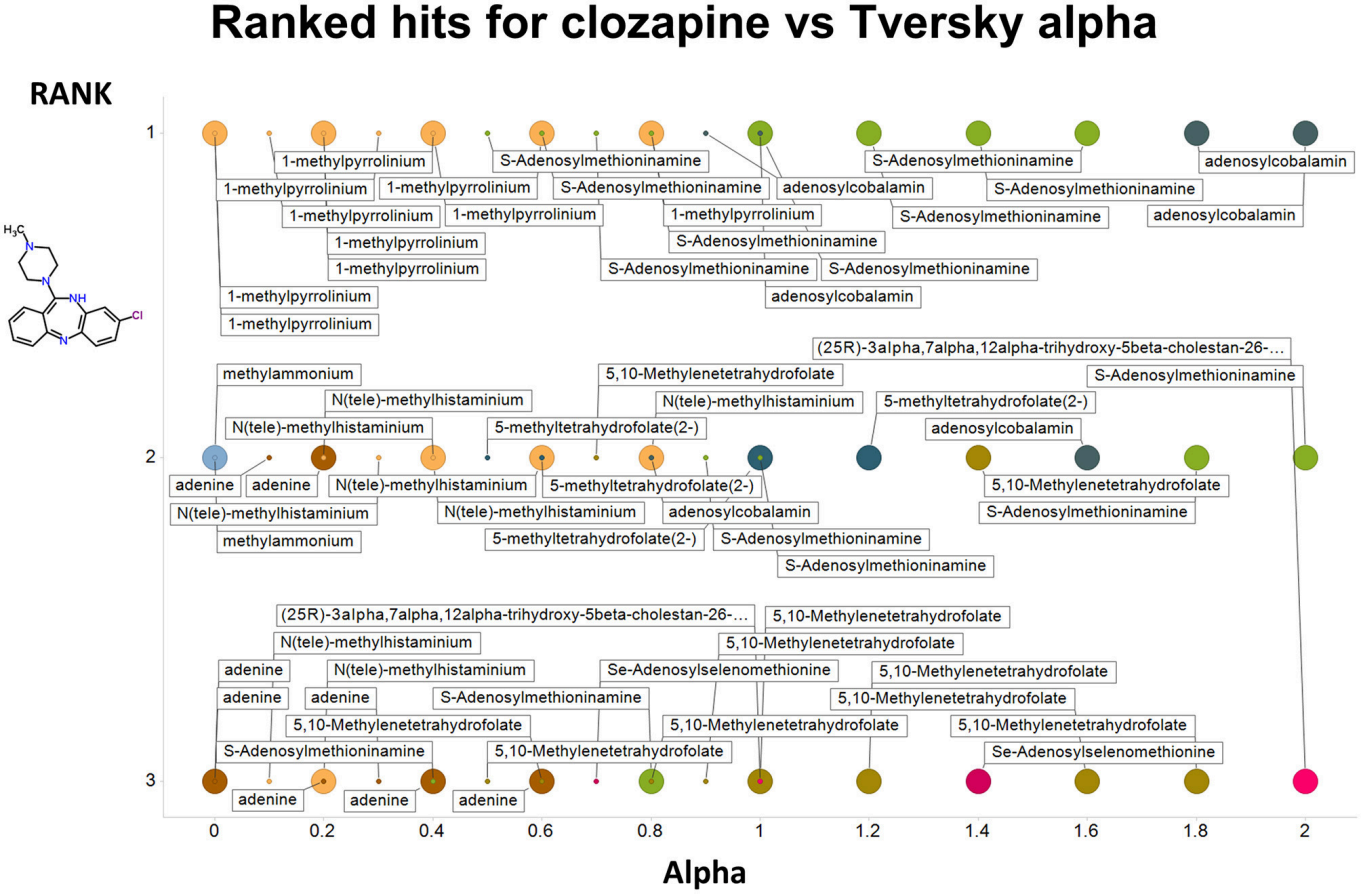
**As Figure 4, save the interrogating molecule is clozapine**.

Figure 2 summarizes visually, via a series of 11 heatmaps, the effects of varying the Tversky α parameter in a comparison of drugs (vertical axes) and endogenites (horizontal axes), using the MACCS166 encoding (Durant et al., 2002), under conditions in which α + β = 1. Obviously there is a very substantial change in the apparent overall similarities of drugs and endogenites, with a strong tendency for greater overall similarities when alpha is closest to zero or 1, and with the similarities in general being considerably greater than the Tanimoto similarities described previously for the MACCS encoding (O'Hagan and Kell, 2015c; O'Hagan et al., 2015; which is the only one we use here). Figure 3 shows the cumulative effect of varying α using the data in Figure 2, which makes even more clear the fact that similarities can be much greater than those observed when Tanimoto is used. Also marked is the fraction of drugs whose largest Tversky similarity to an endogenite exceeds 0.8 (these will appear, with other data, in a secondary plot in **Figure 10**), where it is obvious that again this is a very strong function of α. There is also a clear tendency for the endogenites that are chosen simply to be more complex as α is increased, with (as implied above) CoA derivatives featuring much more than in the cases when α is lower. To this end, Figure 4 shows the similarities of the top 3 metabolites to chlorpromazine at different values of α, while Figure 5 provides similar data to those of Figure 2 for a number of cases of α for conditions in which α + β = 2 (as occurs for the Tanimoto similarity where α = β = 1), and with the cumulative plots equivalent to those for α + β = 1, shown now for α + β = 2, in Figure 6. As for the case in which α + β = 1, the trend is similar, with overall similarities being greatest when α is nearer its extreme values. However, the similarity values are generally much lower than when α + β = 1 (see the much greater extent of blue in the heatmaps in Figure 5, and the ordinate values in Figure 6); indeed it is seen that the Tanimoto coefficient (α = β = 1, α + β = 2), with 90% of drugs showing a TS > 0.5 as before (O'Hagan and Kell, 2015c; O'Hagan et al., 2015), is a poor choice if one is seeking to maximize the apparent similarity between two molecules. Similarly, the nature of the molecules whose similarity to a different interrogatory molecule is greatest also changes significantly with α. This is again illustrated, now for clozapine, in Figure 7. The data for the “top 20” similarities for chlorpromazine and for clozapine are given as Tables S1, S2.

**Figure 8:**
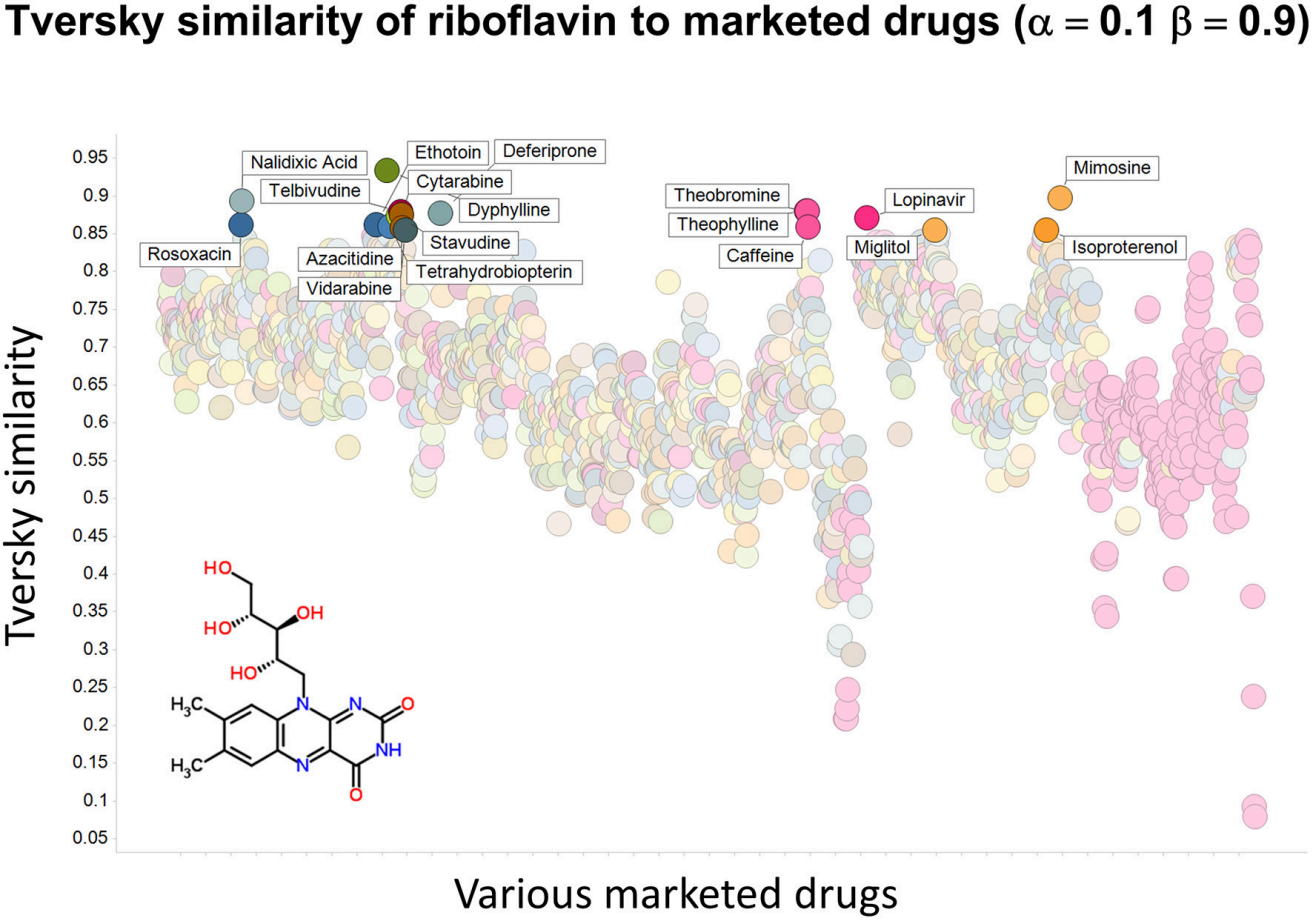
**Tversky similarity (α = 0.1, β = 0.9) of riboflavin to marketed drugs**. Names are given for those with values of 0.85 or greater.

**Figure 9:**
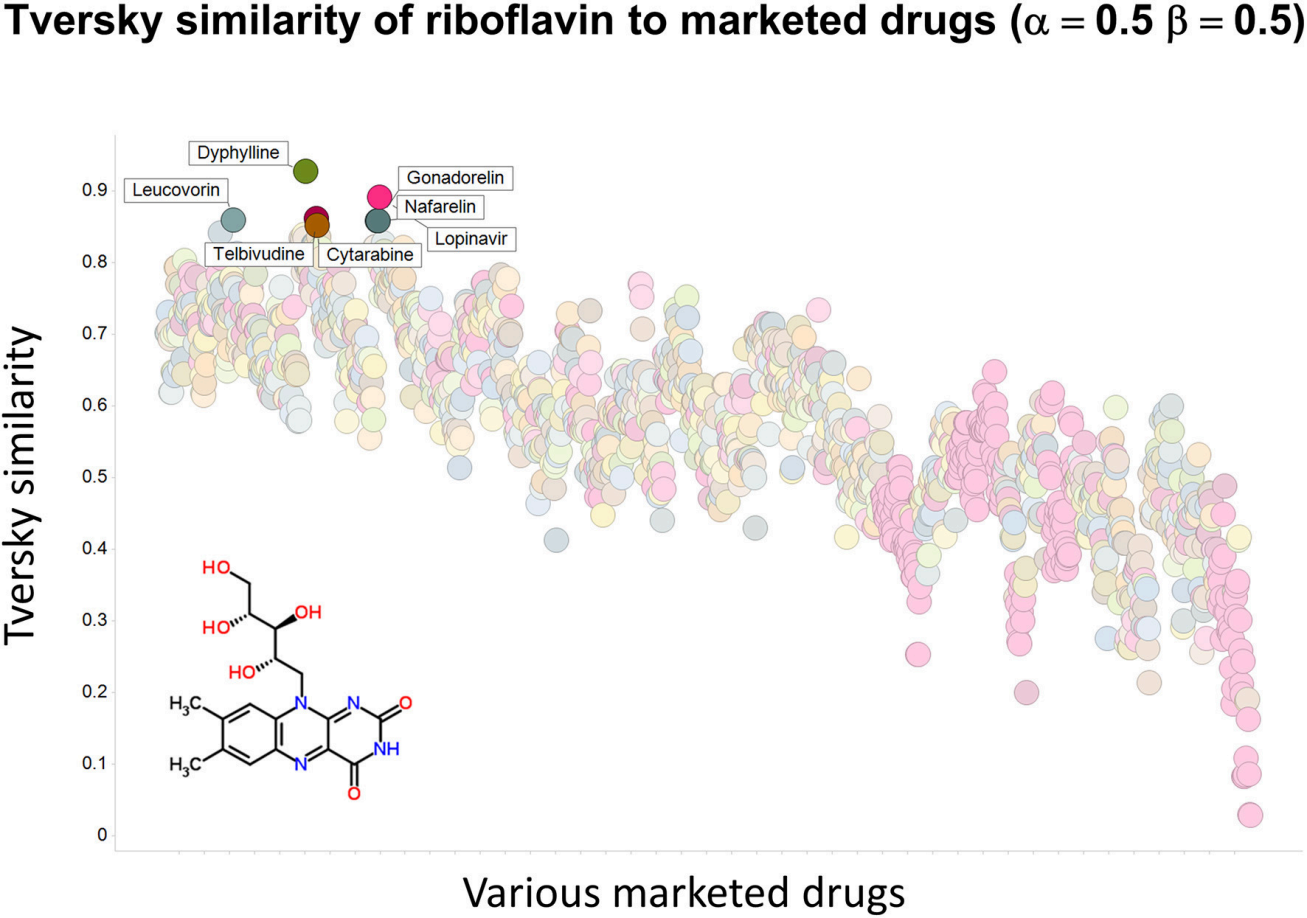
**Tversky similarity (α = 0.5, β = 0.5) of riboflavin to marketed drugs**. Names are given for those with values of 0.85 or greater.

To illustrate that this improved variation in apparent molecular similarity works “both ways,” we use an endogenite, riboflavin, as the interrogating molecule, and assess its similarity to marketed drugs. Figures 8, 9 show the top hits for α = 0.1, β = 0.9, and α = 0.5, β = 0.5, respectively. Obviously, again, not only the typical magnitudes of the Tversky similarity change significantly but so does the rank order of molecules.

**Figure 10:**
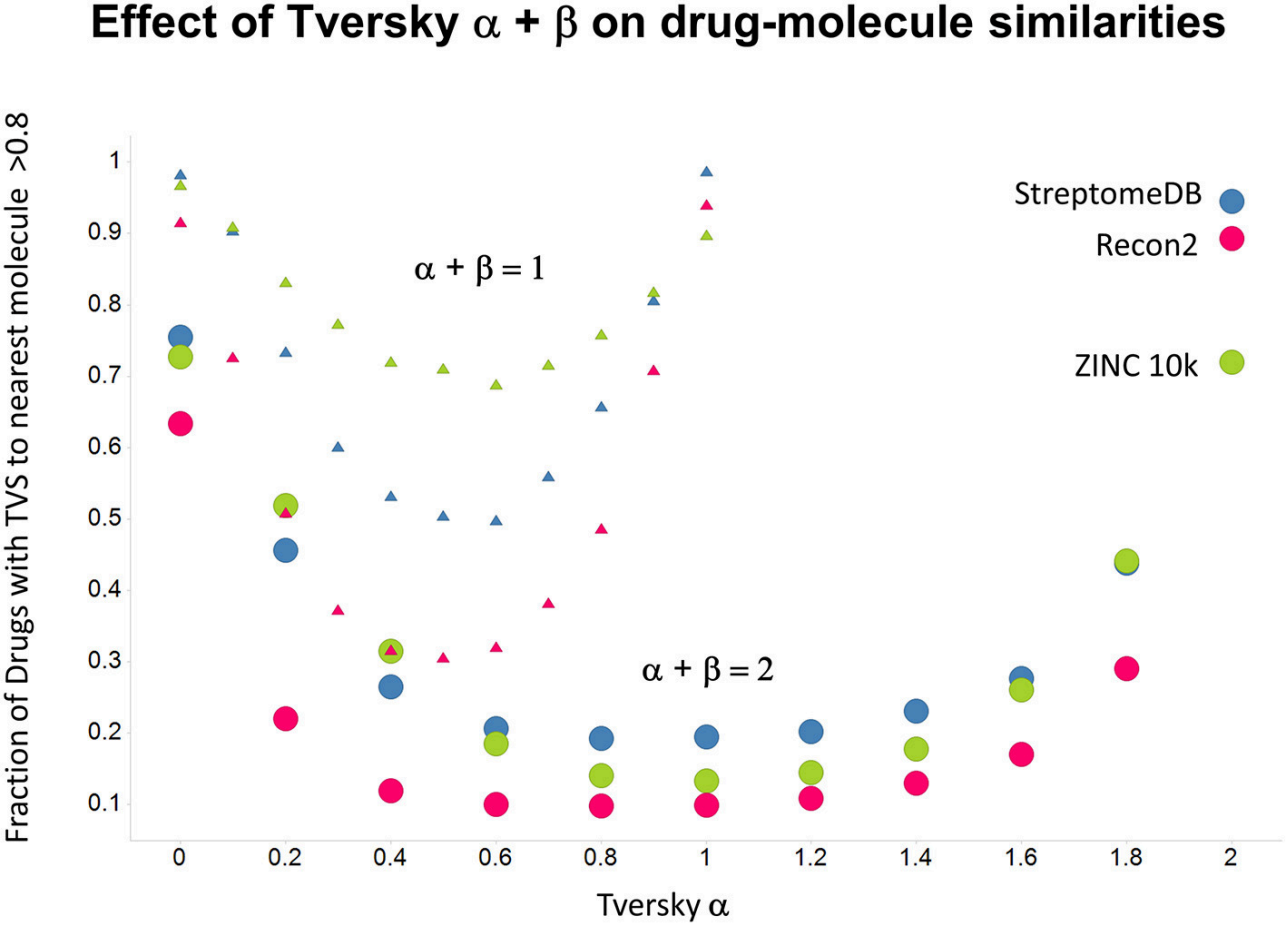
**Fraction of marketed drugs with a Tversky similarity >0.8 to at least one molecule in the stated collections**. The comparison was against Recon2 (1112 molecules), streptome DB (Lucas et al., 2013) (2400 molecules) and a random subset of 10,000 molecules drawn from the ZINC (Irwin and Shoichet, 2005) database. Colors in this and the following three figures are labeled by the points for α = 2.

**Figure 11:**
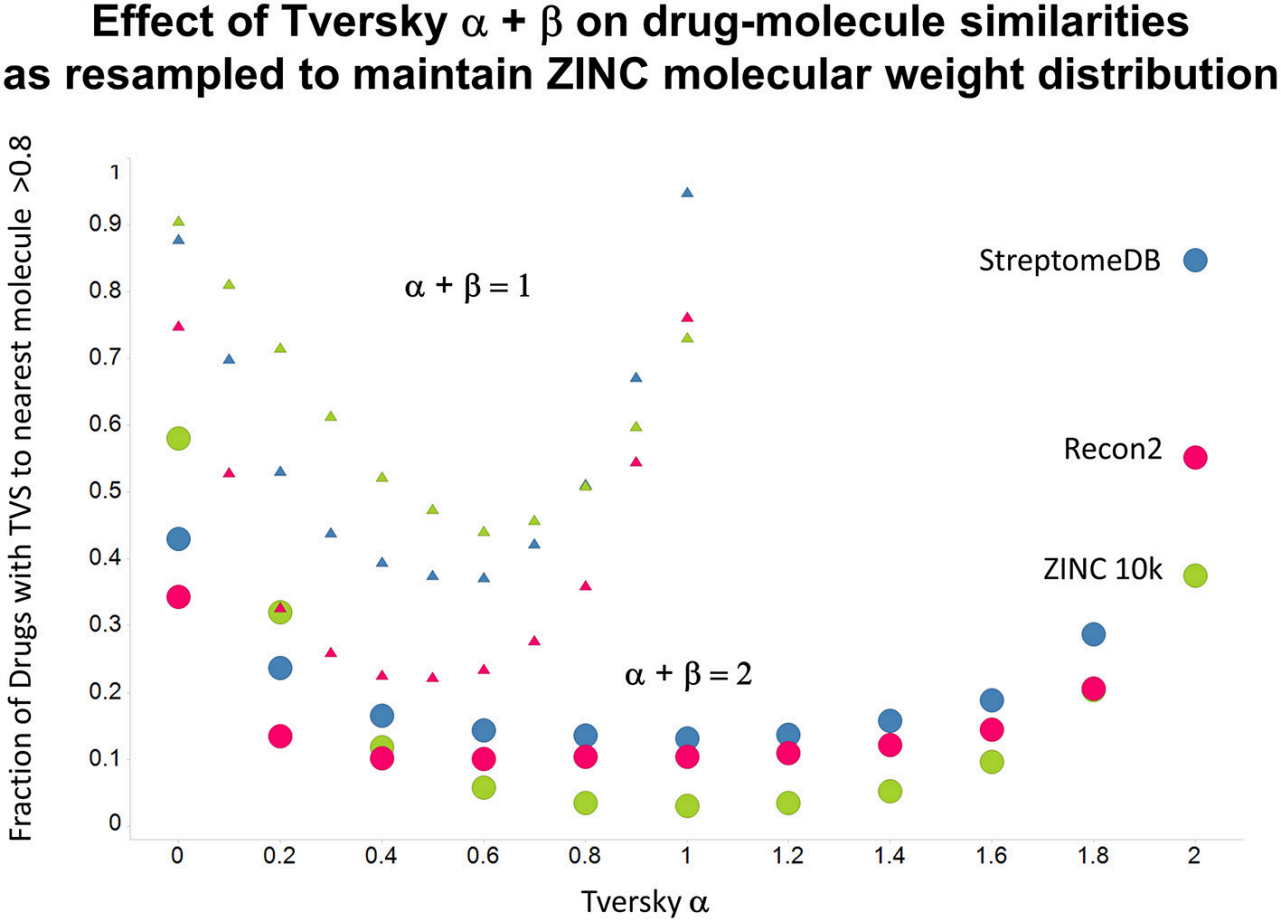
**As Figure 10, but data are subsampled to retain the same MW distrubtion for each class (which is effectively that of ZINC)**.

**Figure 12:**
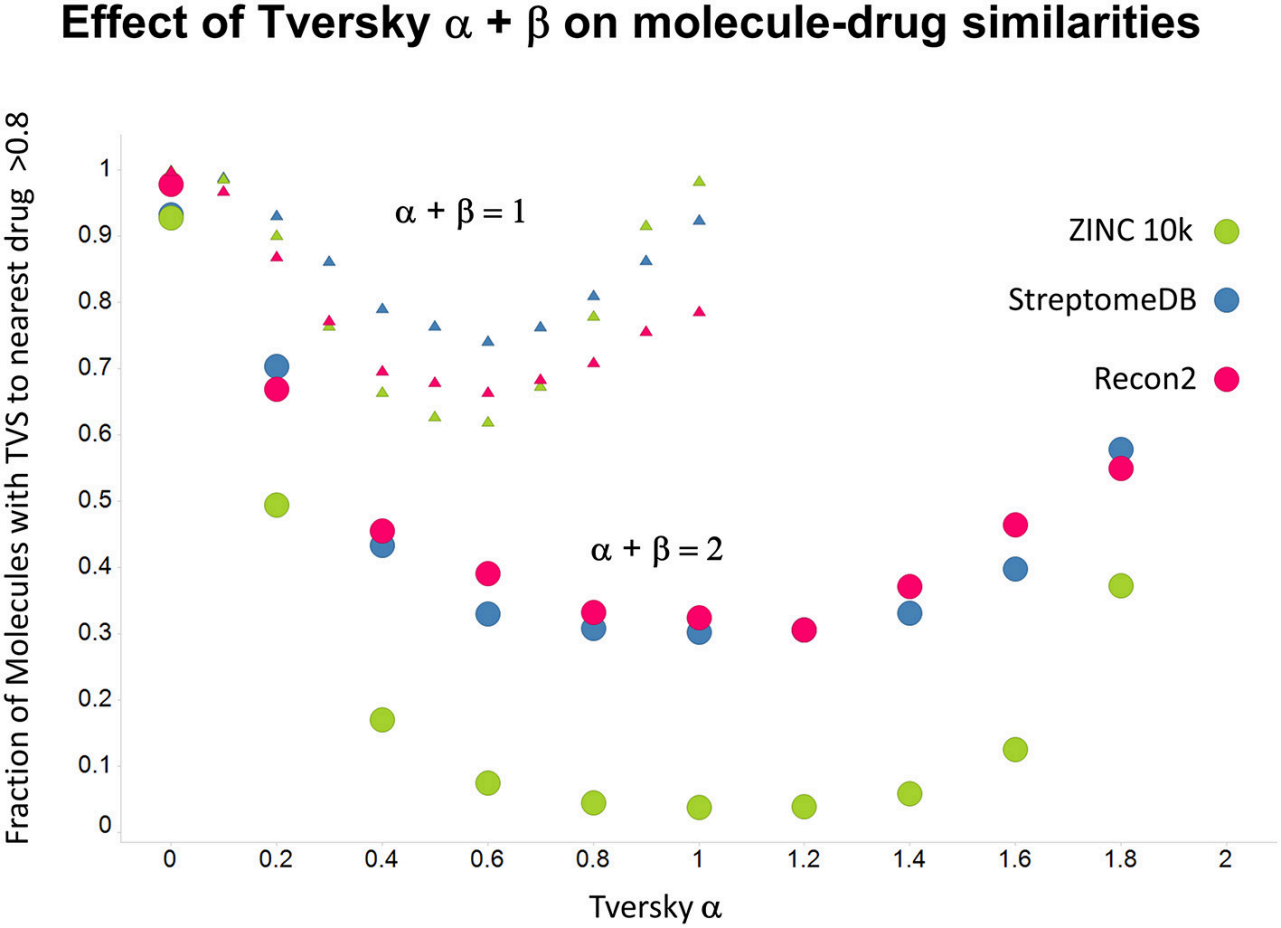
**Fraction of molecules with a Tversky similarity >0.8 to at least one marketed drug in the stated collections**. The comparison was against Recon2 (1112 molecules), streptome DB (Lucas et al., 2013) (2400 molecules) and a random subset of 10,000 molecules drawn from the ZINC (Irwin and Shoichet, 2005) database.

**Figure 13:**
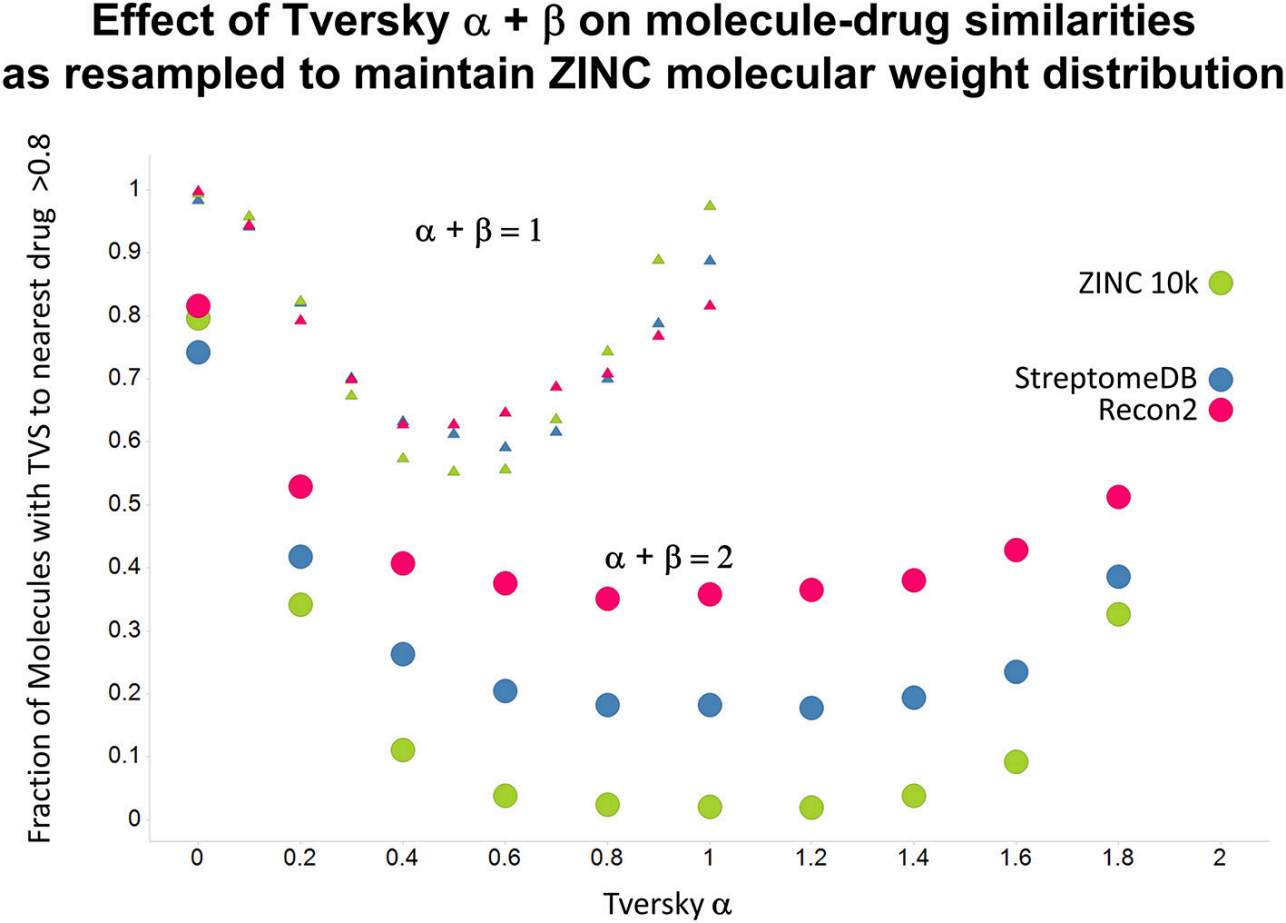
**As Figure 12, but data are subsampled to retain the same MW distrubtion for each class (which is effectively that of ZINC)**.

As shown before (O'Hagan and Kell, 2015c; O'Hagan et al., 2015), the shape of these cumulative plots (Figures 3, 6) of the similarities of marketed drugs to other molecules also depends on the nature of those other molecules. Thus, the overall similarities to marketed drugs were in the order endogenites > natural product library > synthetic chemical library. The question then arises, and this allows a semi-supervised analysis, as to whether there are values of α and β that minimize or maximize these differences. Figure 10 provides a secondary plot of the data shown in Figures 3, 6 for the fraction of drugs exceeding a (somewhat arbitrary) Tversky similarity of 0.8 as a function of α for both α + β = 1 (small symbols) and α + β = 2 (larger symbols). It is clear that both the magnitude *and* the apparent ranking of classes change as a function of the type of library. As before, when α = β = 1 (i.e., Tanimoto similarity), Recon2 metabolites are more like drugs than are natural products and ZINC library members. Figure 10 also shows the same secondary plot for 2400 molecules from StreptomeDB (Lucas et al., 2013; as representative of natural products) and from a subset of 10,000 molecules taken from the ZINC database (Irwin and Shoichet, 2005; Irwin, 2008; Irwin et al., 2012; Sterling and Irwin, 2015). Data for α = β = 1 (Tanimoto similarity) are essentially as previously published (O'Hagan et al., 2015; note that we take random subsets). However, extraordinarily striking differences are seen in the percentage of drugs exceeding a Tversky similarity of 0.8 to the different classes as α and β are varied. Thus, if one wishes to favor the druglikeness of natural products over molecules in ZINC then α + β = 2 is to be preferred, whereas α + β = 1 favors ZINC. We note (as before, O'Hagan et al., 2015) that the molecular weight distributions are not the same for the three classes, with those for ZINC being lowest, and that this could potentially be an issue in that TS favors larger molecules (see above). It is obvious that the varying ranking order of the classes at different values of α and β means that this is not a dominant issue. However, some differences were obtained when we sampled randomly from the classes in a manner that normalized the samples to have the same MW distribution, albeit that this also “clips” those endogenites with high molecular weights (not shown), and these are shown in Figure 11. We also ran the converse query, where the various classes of non-drugs are used to interrogate the list of marketed drugs for apparently similarity, with broadly converse findings (Figures 12, 13).

## Discussion

The general notion of the “similarity” between two or more objects, or their “closeness,” is a complex one (e.g., Johnson and Maggiora, 1990; Rouvray, 1992; Everitt, 1993; Bunke, 1997; Handl et al., 2005; Handl and Knowles, 2007), and this is no less true of molecular similarity (e.g., Hall et al., 1995; Willett et al., 1998; Gasteiger, 2003; Bender and Glen, 2004; Bender et al., 2006; Maldonado et al., 2006; Eckert and Bajorath, 2007; Gallegos-Saliner et al., 2008; Marín et al., 2008; Baldi and Nasr, 2010; Maggiora and Shanmugasundaram, 2011; Maggiora et al., 2014; Medina-Franco and Maggiora, 2014). Here, we confine ourselves to systems in which all the features used are transformed to simple bitstrings that may then be compared. Classical numerical (including chemo) taxonomy (Sneath and Sokal, 1973) gave equal weightings to each binary character, and this is clearly the most unbiased means by which one can make assessments of *overall* similarity. By contrast, a different tradition (e.g., Everitt, 1993; Petrone et al., 2012) asserts that any measurement of a similarity or clustering should be judged solely on its utility, in other words there are usually benefits to the use of a what in statistics is called a “*biased* estimator” (Hastie et al., 2009).

Our previous work comparing endogenites and successful (marketed) drugs showed that they did indeed share similarities, and more so than with the kinds of non-natural molecules common in drug discovery libraries (Dobson P. D. et al., 2009; O'Hagan and Kell, 2015c; O'Hagan et al., 2015). It was also noted that the nature and extent of these similarities could vary significantly with the type of (2D) molecular encoding used. However, in all of that work, the actual bitstring comparisons were based on the use of the Jaccard/Tanimoto similarity coefficient, as is indeed most common in cheminformatics (Willett, 2014). As a single metric, this admits only an unsupervised comparison.

However, the Tanimoto similarity is actually but one member of a larger family of similarity coefficients introduced by Tversky (1977), and it was of interest to see whether the use of a Tversky similarity coefficient Tv(A,B) might provide further information or utility. The Tversky similarity coefficient is indeed occasionally used in cheminformatics (Willett et al., 1998; Chen et al., 2005; Swamidass and Baldi, 2007; Ebalunode et al., 2008; Nasr et al., 2009; Rupp et al., 2009; Senger, 2009; Nicholls et al., 2010; Backman et al., 2011; Geitmann et al., 2011; Berenger et al., 2014; Gan et al., 2014; and also Wang et al., 2007; Wang and Bajorath, 2008), though that used in those papers seems to be based on a different definition from ours, but does not seem to enjoy widespread cheminformatics use. The attraction of Tversky similarities is that they effectively give different weightings to different molecular features, and some of these are likely to be more, and some less, important for understanding the bioactivity or other property of interest. Here we used it in a large-scale comparison of the structures of endogenous human metabolites and marketed drugs. It turned out that variants of the Tversky similarity do indeed provide a much richer harvest of “similar” molecules than do those provided (O'Hagan and Kell, 2015c; O'Hagan et al., 2015) by the standard Tanimoto similarity. The similarities differ both in magnitude and in rank order as α and β and their sum are varied, and thus provide a much broader range of candidate molecules to consider for experimental studies of interest. Being able to incorporate the similarity as part of a surrogate objective function thus allows the use of what amounts to a semi-supervised strategy.

We and others have written before about the potential utility of understanding the “likeness” of individual molecules to those considered representative of particular classes, such as drug-likeness (e.g., Karakoc et al., 2006; Paolini et al., 2006; Bickerton et al., 2012), natural-product-likeness (Ertl et al., 2008; Jayaseelan et al., 2012), or indeed metabolite-likeness (e.g., Cherkasov, 2006; Gupta and Aires-De-Sousa, 2007; Dobson P. D. et al., 2009; Peironcely et al., 2011; Walters, 2012; O'Hagan and Kell, 2015c; O'Hagan et al., 2015). Clearly this depends on the nature of the encoding used, but, as we see here, it can also depend markedly on the metric of similarity, that can be varied via the Tversky α and β parameters.

**Figure 14:**
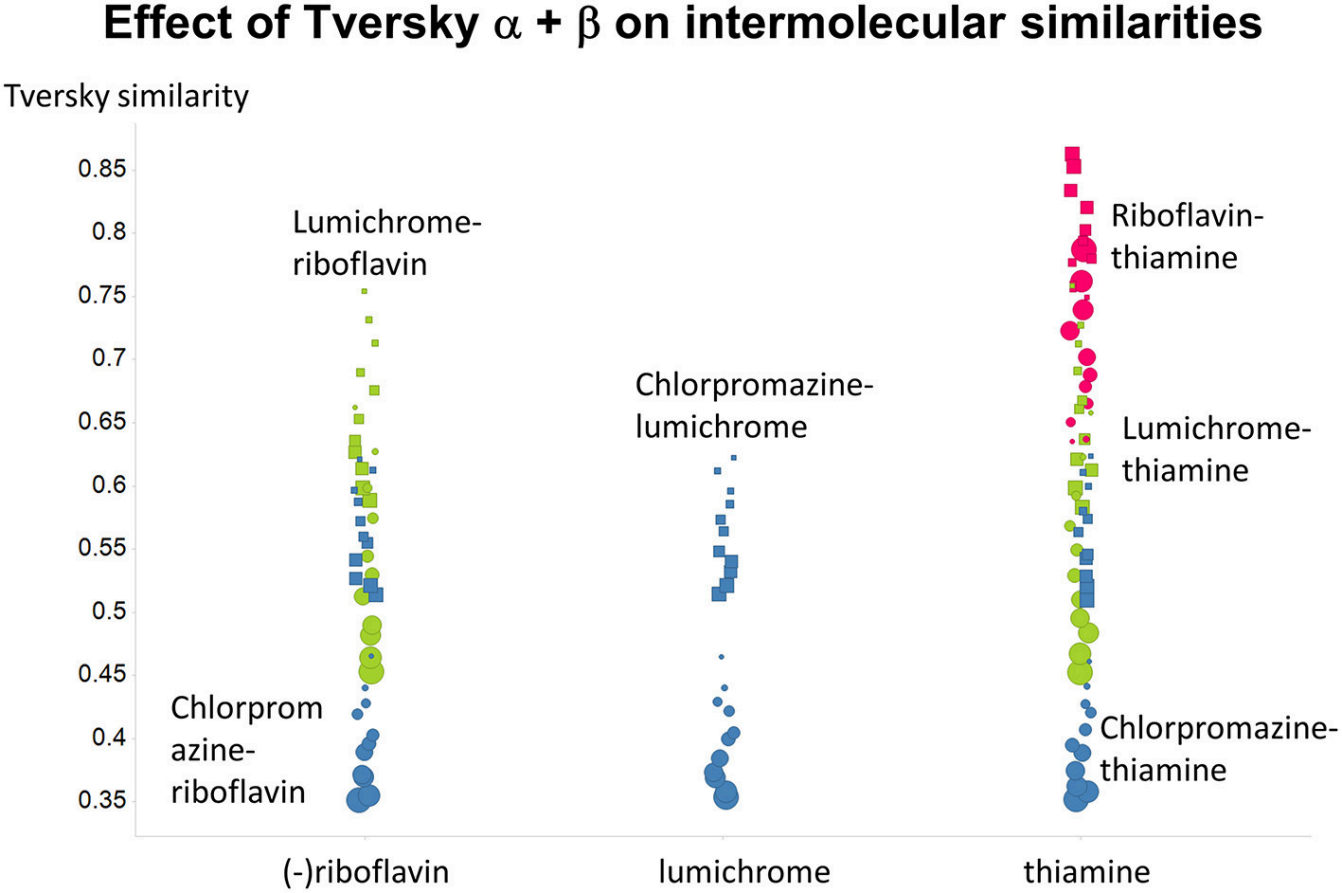
**Variance of Tversky similarities for the molecules that were depicted in Figure 1, as a function of α and β**. Size proportional to α. Squares α + β = 1; circles α + β = 2.

Previously, we found that the shapes of these curves of cumulative similarity differed markedly for different classes of compounds, e.g., when the comparison was made between marketed drugs and natural products or marketed drugs and subsets from drug discovery libraries rather than between drugs and Recon2 (O'Hagan and Kell, 2015c; O'Hagan et al., 2015). It was thus of considerable interest to see how this changed when we used Tversky instead of Taniomoto similarities. Most interestingly, it was not at all the case that the values of α and β favoring drug-likeness were always the greatest for endogenites (as they were for the Tanimoto similarity); particular values could make natural products libraries and ZINC compounds overtake them (Figures 10, 11). Thus it is possible to “tune” the Tversky parameters to favor the kinds of molecules that are most similar to marketed drugs. In a similar vein, the converse can be observed when we run the system “backwards,” interrogating the list of drugs serially with compounds in the three classes (Figures 12–14). Overall, for individual comparisons, the Tversky similarities could easily vary by as much as 0.3 over the ranges of α and β over the range examined here.

Much as our earlier studies (Dobson P. D. et al., 2009; O'Hagan and Kell, 2015c; O'Hagan et al., 2015) had indicated, the more things one varies in even quite an elementary molecular comparison, and even using standard methods, the greater the range of molecular similarities that can become apparent. The present work extends this, by including variants of the comparison metric itself, spreading the Tanimoto similarity to the family of Tversky similarities. The much increased richness of molecular similarity space thereby uncovered, even for just a few interrogations, implies that the Tversky similarities will be of much more use in cheminformatics than their comparatively sparse use to date might imply. We are not yet in a position to recommend specific values of the Tversky parameters; rather we recognize that they simply increase the richness of the molecular space one should take into account when evaluating similarity. As more data emerge it is entirely possible that preferred values of α and β will emerge with them. An obvious extension is to compare the utility of Tversky α and β when different molecular encodings are used.

## Authors information

DK is a Research Professor at the University of Manchester, a role to which he returned full time following a 0.8FTE 5-year secondment at Chief Executive of the Biotechnology and Biological Sciences Research Council. He was previously Director of the Manchester Centre for Integrative Systems Biology (www.mcisb.org). His interests include systems biology, chemical biology, pharmaceutical drug transporters, synthetic biology, cheminformatics, bacterial dormancy, machine learning and iron metabolism. His website is http://dbkgroup.org and he tweets as @dbkell. At Google Scholar his work has been cited more than 33,000 times, with an H-index of 91. SO has a Ph.D. in Chemistry from Warwick University, and following a period in industry is now a Computer Officer at the University of Manchester, specializing in cheminformatics, chemometrics, machine learning and the closed-loop automation of scientific instrumentation.

## Author contributions

DK and SO conceived of the study, participated in its design and coordination and helped to draft the manuscript. SO wrote the workflows. All authors read and approved the final manuscript.

### Conflict of interest statement

The authors declare that the research was conducted in the absence of any commercial or financial relationships that could be construed as a potential conflict of interest.

## Supplementary material

Table S1 **“Top 20” hits for chlorpromazine cpz_hits_new.xlsx**.

Table S2 **“Top 20” hits for clozapine clozapine_hits_new.xlsx for various values of α**.

## References

[B1] Backman T. W. H. Cao Y. Girke T. (2011). ChemMine tools: an online service for analyzing and clustering small molecules. Nucleic Acids Res. 39, W486–W491. 10.1093/nar/gkr320 21576229 PMC3125754

[B2] Balcan M. F. Blum A. (2010). A discriminative model for semi-supervised learning. J. ACM 57, 671–680. 10.1145/1706591.1706599

[B3] Baldi P. Nasr R. (2010). When is chemical similarity significant? The statistical distribution of chemical similarity scores and its extreme values. J. Chem. Inf. Model. 50, 1205–1222. 10.1021/ci100010v 20540577 PMC2914517

[B4] Beisken S. Meinl T. Wiswedel B. De Figueiredo L. F. Berthold M. Steinbeck C.. (2013). KNIME-CDK: Workflow-driven cheminformatics. BMC Bioinformatics 14:257. 10.1186/1471-2105-14-257 24103053 PMC3765822

[B5] Bender A. Glen R. C. (2004). Molecular similarity: a key technique in molecular informatics. Org. Biomol. Chem. 2, 3204–3218. 10.1039/b409813g 15534697

[B6] Bender A. Jenkins J. L. Li Q. L. Adams S. E. Cannon E. O. Glen R. C. (2006). Molecular similarity: advances in methods, applications and validations in virtual screening and qsar. Ann. Rep. Comput. Chem. 2, 141–168. 10.1016/S1574-1400(06)02009-3 PMC7185533 32362803

[B7] Bengio Y. (2009). Learning deep architectures for AI. Found Trends Mach. Learn 2, 1–127. 10.1561/2200000006

[B8] Berenger F. Voet A. Lee X. Y. Zhang K. Y. J. (2014). A rotation-translation invariant molecular descriptor of partial charges and its use in ligand-based virtual screening. J. Cheminform. 6:23. 10.1186/1758-2946-6-23 24887178 PMC4030740

[B9] Berthold M. R. Cebron N. Dill F. Gabriel T. R. Kötter T. Meinl T. (2008). KNIME: the Konstanz Information Miner, in Data Analysis, Machine Learning and Applications, eds Preisach C. Burkhardt H. Schmidt-Thieme L. Decker R. (Berlin: Springer), 319–326.

[B10] Bickerton G. R. Paolini G. V. Besnard J. Muresan S. Hopkins A. L. (2012). Quantifying the chemical beauty of drugs. Nat. Chem. 4, 90–98. 10.1038/nchem.1243 22270643 PMC3524573

[B11] Broadhurst D. Kell D. B. (2006). Statistical strategies for avoiding false discoveries in metabolomics and related experiments. Metabolomics 2, 171–196. 10.1007/s11306-006-0037-z

[B12] Broomhead D. S. Lowe D. (1988). Multivariable function interpolation and adaptive networks. Complex Syst. 2, 321–355.

[B13] Bunke H. (1997). On a relation between graph edit distance and maximum common subgraph. Patt. Recogn. Lett. 18, 689–694. 10.1016/S0167-8655(97)00060-3

[B14] Caldinelli L. Molla G. Bracci L. Lelli B. Pileri S. Cappelletti P.. (2010). Effect of ligand binding on human D-amino acid oxidase: implications for the development of new drugs for schizophrenia treatment. Protein Sci. 19, 1500–1512. 10.1002/pro.429 20521334 PMC2923503

[B15] César-Razquin A. Snijder B. Frappier-Brinton T. Isserlin R. Gyimesi G. Bai X.. (2015). A call for systematic research on solute carriers. Cell 162, 478–487. 10.1016/j.cell.2015.07.022 26232220

[B16] Chapelle O. Schölkopf B. Zien A. (eds.). (2010). Semi-Supervised Learning. Cambridge, MA: MIT Press.

[B17] Chen J. Swamidass S. J. Dou Y. Bruand J. Baldi P. (2005). ChemDB: a public database of small molecules and related chemoinformatics resources. Bioinformatics 21, 4133–4139. 10.1093/bioinformatics/bti683 16174682

[B18] Cherkasov A. (2006). Can ‘Bacterial-Metabolite-Likeness’ model improve odds of ‘*in silico*’ antibiotic discovery? J. Chem. Inf. Model. 46, 1214–1222. 10.1021/ci050480j 16711741

[B19] Cherkasov A. Muratov E. N. Fourches D. Varnek A. Baskin I. I. Cronin M.. (2014). QSAR modeling: where have you been? where are you going to? J. Med. Chem. 57, 4977–5010. 10.1021/jm4004285 24351051 PMC4074254

[B20] Demiriz A. Bennett K. Embrechts M. J. (1999). Semi-supervised clustering using genetic algorithms, in Intelligent engineering systems through artificial neural networks, eds Dagli C.H. Buczak A.L. Ghosh J. Embrechts M.J. Ersoy O. (New York, NY: ASME Press), 809–814.

[B21] Dixon S. L. Koehler R. T. (1999). The hidden component of size in two-dimensional fragment descriptors: side effects on sampling in bioactive libraries. J. Med. Chem. 42, 2887–2900. 10.1021/jm980708c 10425098

[B22] Dobson P. D. Kell D. B. (2008). Carrier-mediated cellular uptake of pharmaceutical drugs: an exception or the rule? Nat. Rev. Drug. Discov. 7, 205–220. 10.1038/nrd2438 18309312

[B23] Dobson P. D. Patel Y. Kell D. B. (2009). “Metabolite-likeness” as a criterion in the design and selection of pharmaceutical drug libraries. Drug Discov. Today 14, 31–40. 10.1016/j.drudis.2008.10.011 19049901

[B24] Dobson P. Lanthaler K. Oliver S. G. Kell D. B. (2009). Implications of the dominant role of cellular transporters in drug uptake. Curr. Top. Med. Chem. 9, 163–184. 10.2174/156802609787521616 19200003

[B25] Durant J. L. Leland B. A. Henry D. R. Nourse J. G. (2002). Reoptimization of MDL keys for use in drug discovery. J. Chem. Inf. Comput. Sci. 42, 1273–1280. 10.1021/ci010132r 12444722

[B26] Ebalunode J. O. Ouyang Z. Liang J. Zheng W. (2008). Novel approach to structure-based pharmacophore search using computational geometry and shape matching techniques. J. Chem. Inf. Model. 48, 889–901. 10.1021/ci700368p 18396858

[B27] Ecker G. Chiba P. (eds.). (2009). Transporters as Drug Carriers: Structure, Function, Substrates. Weinheim: Wiley/VCH.

[B28] Ecker G. F. (2014). Transmembrane drug transporter - taxonomy, assays, and their role in drug discovery. Drug Discov. Today Technol. 12, e35–e36. 10.1016/j.ddtec.2014.04.002 25027373

[B29] Eckert H. Bajorath J. (2007). Molecular similarity analysis in virtual screening: foundations, limitations and novel approaches. Drug Discov. Today 12, 225–233. 10.1016/j.drudis.2007.01.011 17331887

[B30] Erhan D. Bengio Y. Courville A. Manzagol P. A. Vincent P. Bengio S. (2010). Why does unsupervised pre-training help deep learning? J. Mach. Learn. Res. 11, 625–660.

[B31] Ertl P. Roggo S. Schuffenhauer A. (2008). Natural product-likeness score and its application for prioritization of compound libraries. J. Chem. Inf. Model. 48, 68–74. 10.1021/ci700286x 18034468

[B32] Everitt B. S. (1993). Cluster Analysis. London: Edward Arnold.

[B33] Flower D. R. (1998). On the properties of bit string-based measures of chemical similarity. J. Chem. Inf. Comp. Sci. 38, 379–386. 10.1021/ci970437z

[B34] Fromm M. F. Kim R. B. (eds.). (2011). Drug Transporters. Berlin: Springer.

[B35] Gabay S. Harris S. R. (1965). Studies of flavin adenine dinucleotide-requiring enzymes and phenothiazines-I. interactions of chlorpromazine and D-amino acid oxidase. Biochem. Pharmacol. 14, 17–26. 10.1016/0006-2952(65)90053-5 14286097

[B36] Gallegos-Saliner A. Poater A. Jeliazkova N. Patlewicz G. Worth A. P. (2008). Toxmatch–a chemical classification and activity prediction tool based on similarity measures. Regul. Toxicol. Pharmacol. 52, 77–84. 10.1016/j.yrtph.2008.05.012 18617309

[B37] Gan S. Cosgrove D. A. Gardiner E. J. Gillet V. J. (2014). Investigation of the use of spectral clustering for the analysis of molecular data. J. Chem. Inf. Model. 54, 3302–3319. 10.1021/ci500480b 25379955

[B38] Gasteiger J. (ed.). (2003). Handbook of Chemoinformatics: From Data to Knowledge. Weinheim: Wiley/VCH.

[B39] Geitmann M. Elinder M. Seeger C. Brandt P. De Esch I. J. P. Danielson U. H.. (2011). Identification of a novel scaffold for allosteric inhibition of wild type and drug resistant HIV-1 reverse transcriptase by fragment library screening. J. Med. Chem. 54, 699–708. 10.1021/jm1010513 21207961

[B40] Giacomini K. M. Huang S. M. (2013). Transporters in drug development and clinical pharmacology. Clin. Pharmacol. Ther. 94, 3–9. 10.1038/clpt.2013.86 23778703

[B41] Giacomini K. M. Huang S. M. Tweedie D. J. Benet L. Z. Brouwer K. L. Chu X.. (2010). Membrane transporters in drug development. Nat. Rev. Drug Discov. 9, 215–236. 10.1038/nrd3028 20190787 PMC3326076

[B42] Gründemann D. (2012). The ergothioneine transporter controls and indicates ergothioneine activity–a review. Prev. Med. 54(Suppl.), S71–S74. 10.1016/j.ypmed.2011.12.001 22182480

[B43] Gründemann D. Harlfinger S. Golz S. Geerts A. Lazar A. Berkels R.. (2005). Discovery of the ergothioneine transporter. Proc. Natl. Acad. Sci. U.S.A. 102, 5256–5261. 10.1073/pnas.0408624102 15795384 PMC555966

[B44] Gupta S. Aires-De-Sousa J. (2007). Comparing the chemical spaces of metabolites and available chemicals: models of metabolite-likeness. Mol. Divers. 11, 23–36. 10.1007/s11030-006-9054-0 17447158

[B45] Hall L. H. Kier L. B. Brown B. B. (1995). Molecular similarity based on novel atom-type electrotopological state indexes. J. Chem. Inf. Comp. Sci. 35, 1074–1080. 10.1021/ci00028a019

[B46] Handl J. Knowles J. (2006). Semi-supervised feature selection via multiobjective optimization, in IEEE Proceedings of International Joint Conference on Neural Network (Vancouver, BC), 3319–3326.

[B47] Handl J. Knowles J. (2007). An evolutionary approach to multiobjective clustering. IEEE Trans. Evol. Comput. 11, 56–76. 10.1109/TEVC.2006.877146

[B48] Handl J. Knowles J. Kell D. B. (2005). Computational cluster validation in post-genomic data analysis. Bioinformatics 21, 3201–3212. 10.1093/bioinformatics/bti517 15914541

[B49] Hastie T. Tibshirani R. Friedman J. (2009). The Elements Of Statistical Learning: Data Mining, Inference and Prediction, 2nd Edn. Berlin: Springer-Verlag.

[B50] Hediger M. A. Clémencon B. Burrier R. E. Bruford E. A. (2013). The ABCs of membrane transporters in health and disease (SLC series): Introduction. Mol. Aspects Med. 34, 95–107. 10.1016/j.mam.2012.12.009 23506860 PMC3853582

[B51] Herrgård M. J. Swainston N. Dobson P. Dunn W. B. Arga K. Y. Arvas M.. (2008). A consensus yeast metabolic network obtained from a community approach to systems biology. Nat. Biotechnol. 26, 1155–1160. 10.1038/nbt1492 18846089 PMC4018421

[B52] Hinton G. E. (2007). Learning multiple layers of representation. Trends Cogn. Sci. 11, 428–434. 10.1016/j.tics.2007.09.004 17921042

[B53] Hinton G. E. Osindero S. Teh Y. W. (2006). A fast learning algorithm for deep belief nets. Neural Comput. 18, 1527–1554. 10.1162/neco.2006.18.7.1527 16764513

[B54] Hinton G. E. Salakhutdinov R. R. (2006). Reducing the dimensionality of data with neural networks. Science 313, 504–507. 10.1126/science.1127647 16873662

[B55] Holliday J. D. Hu C. Y. Willett P. (2002). Grouping of coefficients for the calculation of inter-molecular similarity and dissimilarity using 2D fragment bit-strings. Comb. Chem. High Throughput Screen. 5, 155–166. 10.2174/1386207024607338 11966424

[B56] Hotelling H. (1933). Analysis of a complex of statistical variables into principal components. J. Educ. Psychol. 24, 417–441. 10.1037/h0071325

[B57] Irwin J. J. (2008). Using ZINC to acquire a virtual screening library. Curr Protoc. Bioinformatics Chapter 14, Unit 14.16. 10.1002/0471250953.bi1406s22 18551414

[B58] Irwin J. J. Shoichet B. K. (2005). ZINC–a free database of commercially available compounds for virtual screening. J. Chem. Inf. Model. 45, 177–182. 10.1021/ci049714+ 15667143 PMC1360656

[B59] Irwin J. J. Sterling T. Mysinger M. M. Bolstad E. S. Coleman R. G. (2012). ZINC: a free tool to discover chemistry for biology. J. Chem. Inf. Model. 52, 1757–1768. 10.1021/ci3001277 22587354 PMC3402020

[B60] Ishikawa T. Kim R. B. König J. (eds.). (2013). Pharmacogenomics of Human Drug Transporters: Clinical Impacts. New York, NY: Wiley.

[B61] Iwana S. Kawazoe T. Park H. K. Tsuchiya K. Ono K. Yorita K.. (2008). Chlorpromazine oligomer is a potentially active substance that inhibits human D-amino acid oxidase, product of a susceptibility gene for schizophrenia. J. Enzyme Inhib. Med. Chem. 23, 901–911. 10.1080/14756360701745478 18615285

[B62] Iwasa S. Tabara H. Song Z. Nakabayashi M. Yokoyama Y. Fukushima T. (2011). Inhibition of D-amino acid oxidase activity by antipsychotic drugs evaluated by a fluorometric assay using D-kynurenine as substrate. Yakugaku Zasshi 131, 1111–1116. 10.1248/yakushi.131.1111 21720142

[B63] Jayaseelan K. V. Moreno P. Truszkowski A. Ertl P. Steinbeck C. (2012). Natural product-likeness score revisited: an open-source, open-data implementation. BMC Bioinformatics 13:106. 10.1186/1471-2105-13-106 22607271 PMC3436723

[B64] Johnson M. A. Maggiora G. M. (eds.). (1990). Concepts and Applications of Molecular Similarity. New York, NY: Wiley.

[B65] Karakoc E. Sahinalp S. C. Cherkasov A. (2006). Comparative QSAR- and fragments distribution analysis of drugs, druglikes, metabolic substances, and antimicrobial compounds. J. Chem. Inf. Model. 46, 2167–2182. 10.1021/ci0601517 16995747

[B66] Kell D. B. (2013). Finding novel pharmaceuticals in the systems biology era using multiple effective drug targets, phenotypic screening, and knowledge of transporters: where drug discovery went wrong and how to fix it. FEBS J. 280, 5957–5980. 10.1111/febs.12268 23552054

[B67] Kell D. B. (2015a). The transporter-mediated cellular uptake of pharmaceutical drugs is based on their metabolite-likeness and not on their bulk biophysical properties: Towards a systems pharmacology. Perspect. Sci. 6, 66–83. 10.1016/j.pisc.2015.06.004

[B68] Kell D. B. (2015b). What would be the observable consequences if phospholipid bilayer diffusion of drugs into cells is negligible? Trends Pharmacol. Sci. 36, 15–21. 25458537 10.1016/j.tips.2014.10.005

[B69] Kell D. B. (2016a, August 11). How drugs pass through biological cell membranes—a paradigm shift in our understanding? Beilstein Magazine 2.

[B70] Kell D. B. (2016b). Implications of endogenous roles of transporters for drug discovery: hitchhiking and metabolite-likeness. Nat. Rev. Drug Discov. 15, 143–144. 10.1038/nrd.2015.44 26837595

[B71] Kell D. B. Dobson P. D. (2009). The cellular uptake of pharmaceutical drugs is mainly carrier-mediated and is thus an issue not so much of biophysics but of systems biology, in Proc Int Beilstein Symposium on Systems Chemistry, eds Hicks M. G. Kettner C. (Berlin: Logos Verlag), 149–168. Available online at: http://www.beilstein-institut.de/download/628/09_kell.pdf

[B72] Kell D. B. Dobson P. D. Bilsland E. Oliver S. G. (2013). The promiscuous binding of pharmaceutical drugs and their transporter-mediated uptake into cells: what we (need to) know and how we can do so. Drug Discov. Today 18, 218–239. 10.1016/j.drudis.2012.11.008 23207804

[B73] Kell D. B. Dobson P. D. Oliver S. G. (2011). Pharmaceutical drug transport: the issues and the implications that it is essentially carrier-mediated only. Drug Discov. Today 16, 704–714. 10.1016/j.drudis.2011.05.010 21624498

[B74] Kell D. B. Goodacre R. (2014). Metabolomics and systems pharmacology: why and how to model the human metabolic network for drug discovery. Drug Discov. Today 19, 171–182. 10.1016/j.drudis.2013.07.014 23892182 PMC3989035

[B75] Kell D. B. Oliver S. G. (2014). How drugs get into cells: tested and testable predictions to help discriminate between transporter-mediated uptake and lipoidal bilayer diffusion. Front. Pharmacol. 5:231. 10.3389/fphar.2014.00231 25400580 PMC4215795

[B76] Kell D. B. Swainston N. Pir P. Oliver S. G. (2015). Membrane transporter engineering in industrial biotechnology and whole-cell biocatalysis. Trends Biotechnol. 33, 237–246. 10.1016/j.tibtech.2015.02.001 25746161

[B77] Kingma D. P. Rezendey D. J. Mohamedy S. Welling M. (2014). Semi-supervised learning with deep generative models. Proc. Adv. Neural Inf. Proc. 27, 3581–3589.

[B78] Koepsell H. (2013). The SLC22 family with transporters of organic cations, anions and zwitterions. Mol. Aspects Med. 34, 413–435. 10.1016/j.mam.2012.10.010 23506881

[B79] Landrum G. A. Stiefl N. (2012). Is that a scientific publication or an advertisement? Reproducibility, source code and data in the computational chemistry literature. Future Med. Chem. 4, 1885–1887. 10.4155/fmc.12.160 23088269

[B80] Landrum G. Lewis R. Palmer A. Stiefl N. Vulpetti A. (2011). Making sure there's a “give” associated with the “take”: producing and using open-source software in big pharma. J. Cheminform. 3:O3 10.1186/1758-2946-3-s1-o3

[B81] Lanthaler K. Bilsland E. Dobson P. D. Moss H. J. Pir P. Kell D. B.. (2011). Genome-wide assessment of the carriers involved in the cellular uptake of drugs: a model system in yeast. BMC Biol. 9:70. 10.1186/1741-7007-9-70 22023736 PMC3280192

[B82] Law V. Knox C. Djoumbou Y. Jewison T. Guo A. C. Liu Y.. (2014). DrugBank 4.0: shedding new light on drug metabolism. Nucleic Acids Res. 42, D1091–D1097. 10.1093/nar/gkt1068 24203711 PMC3965102

[B83] Lecun Y. Bengio Y. Hinton G. (2015). Deep learning. Nature 521, 436–444. 10.1038/nature14539 26017442

[B84] Lucas X. Senger C. Erxleben A. Grüning B. A. Doring K. Mosch J.. (2013). StreptomeDB: a resource for natural compounds isolated from *Streptomyces* species. Nucleic Acids Res. 41, D1130–D1136. 10.1093/nar/gks1253 23193280 PMC3531085

[B85] Maggiora G. M. Shanmugasundaram V. (2011). Molecular similarity measures. Methods Mol. Biol. 672, 39–100. 10.1007/978-1-60761-839-3_2 20838964

[B86] Maggiora G. Vogt M. Stumpfe D. Bajorath J. (2014). Molecular similarity in medicinal chemistry. J. Med. Chem. 57, 3186–3204. 10.1021/jm401411z 24151987

[B87] Maldonado A. G. Doucet J. P. Petitjean M. Fan B. T. (2006). Molecular similarity and diversity in chemoinformatics: from theory to applications. Mol. Divers. 10, 39–79. 10.1007/s11030-006-8697-1 16404528

[B88] Marín R. M. Aguirre N. F. Daza E. E. (2008). Graph theoretical similarity approach to compare molecular electrostatic potentials. J. Chem. Inf. Model. 48, 109–118. 10.1021/ci7001878 18166018

[B89] Mazanetz M. P. Marmon R. J. Reisser C. B. T. Morao I. (2012). Drug discovery applications for KNIME: an open source data mining platform. Curr. Top. Med. Chem. 12, 1965–1979. 10.2174/156802612804910331 23110532

[B90] Medina-Franco J. L. Maggiora G. M. (2014). Molecular similarity analysis, in Chemoinformatics for Drug Discovery, ed Bajorath J. (Hoboken, NJ: Wiley), 343–399.

[B91] Mendes P. Oliver S. G. Kell D. B. (2015). Fitting transporter activities to cellular drug concentrations and fluxes: why the bumblebee can fly. Trends Pharmacol. Sci. 36, 710–723. 10.1016/j.tips.2015.07.006 26538313 PMC4642801

[B92] Moody J. Darken C. (1989). Fast learning in networks of locally-tuned processing units. Neural Comput. 1, 281–294. 10.1162/neco.1989.1.2.281

[B93] Nasr R. J. Swamidass S. J. Baldi P. F. (2009). Large scale study of multiple-molecule queries. J. Cheminform. 1:7. 10.1186/1758-2946-1-7 20298525 PMC3225883

[B94] Neal M. J. Goodacre R. Kell D. B. (1994). On the analysis of pyrolysis mass spectra using artificial neural networks. Individual input scaling leads to rapid learning, in Proceedings of the World Congress on Neural Networks: International Neural Network Society (San Diego), 318–323.

[B95] Nicholls A. Mcgaughey G. B. Sheridan R. P. Good A. C. Warren G. Mathieu M.. (2010). Molecular shape and medicinal chemistry: a perspective. J. Med. Chem. 53, 3862–3886. 10.1021/jm900818s 20158188 PMC2874267

[B96] Nigam S. K. (2015). What do drug transporters really do? Nat. Rev. Drug Discov. 14, 29–44. 10.1038/nrd4461 25475361 PMC4750486

[B97] O'Hagan S. Kell D. B. (2015a). The apparent permeabilities of Caco-2 cells to marketed drugs: magnitude, and independence from both biophysical properties and endogenite similarities PeerJ. 3:E1405. 10.7717/peerj.1405 26618081 PMC4655101

[B98] O'Hagan S. Kell D. B. (2015b). Software review: the KNIME workflow environment and its applications in Genetic Programming and machine learning. Genetic Progr. Evol. Mach. 16, 387–391. 10.1007/s10710-015-9247-3

[B99] O'Hagan S. Kell D. B. (2015c). Understanding the foundations of the structural similarities between marketed drugs and endogenous human metabolites. Front. Pharmacol. 6:105. 10.3389/fphar.2015.00105 26029108 PMC4429554

[B100] O'Hagan S. Swainston N. Handl J. Kell D. B. (2015). A ‘rule of 0.5’ for the metabolite-likeness of approved pharmaceutical drugs. Metabolomics 11, 323–339. 10.1007/s11306-014-0733-z 25750602 PMC4342520

[B101] Palsson B. Ø. (2015). Systems Biology: Constraint-Based Reconstruction and Analysis. Cambridge: Cambridge University Press.

[B102] Paolini G. V. Shapland R. H. Van Hoorn W. P. Mason J. S. Hopkins A. L. (2006). Global mapping of pharmacological space. Nat. Biotechnol. 24, 805–815. 10.1038/nbt1228 16841068

[B103] Peironcely J. E. Reijmers T. Coulier L. Bender A. Hankemeier T. (2011). Understanding and classifying metabolite space and metabolite-likeness. PLoS ONE 6:e28966. 10.1371/journal.pone.0028966 22194963 PMC3237584

[B104] Pelliccione N. Pinto J. Huang Y. P. Rivlin R. S. (1983). Accelerated development of riboflavin deficiency by treatment with chlorpromazine. Biochem. Pharmacol. 32, 2949–2953. 10.1016/0006-2952(83)90401-X 6626265

[B105] Petrone P. M. Simms B. Nigsch F. Lounkine E. Kutchukian P. Cornett A.. (2012). Rethinking molecular similarity: comparing compounds on the basis of biological activity. ACS Chem. Biol. 7, 1399–1409. 10.1021/cb3001028 22594495

[B106] Pinto J. Huang Y. P. Rivlin R. S. (1981). Inhibition of riboflavin metabolism in rat tissues by chlorpromazine, imipramine, and amitriptyline. J. Clin. Invest. 67, 1500–1506. 10.1172/JCI110180 6262379 PMC370718

[B107] Riniker S. Fechner N. Landrum G. A. (2013). Heterogeneous classifier fusion for ligand-based virtual screening: or, how decision making by committee can be a good thing. J. Chem. Inf. Model. 53, 2829–2836. 10.1021/ci400466r 24171408

[B108] Riniker S. Landrum G. A. (2013). Open-source platform to benchmark fingerprints for ligand-based virtual screening. J. Cheminform. 5:26. 10.1186/1758-2946-5-26 23721588 PMC3686626

[B109] Riniker S. Wang Y. Jenkins J. L. Landrum G. A. (2014). Using information from historical high-throughput screens to predict active compounds. J. Chem. Inf. Model. 54, 1880–1891. 10.1021/ci500190p 24933016

[B110] Rouvray D. H. (1992). Definition and role of similarity concepts in the chemical and physical sciences. J. Chem. Inf. Comp. Sci. 32, 580–586. 10.1021/ci00010a002

[B111] Rupp M. Schneider P. Schneider G. (2009). Distance phenomena in high-dimensional chemical descriptor spaces: consequences for similarity-based approaches. J. Comput. Chem. 30, 2285–2296. 19266481 10.1002/jcc.21218

[B112] Ruusmann V. Sild S. Maran U. (2014). QSAR DataBank - an approach for the digital organization and archiving of QSAR model information. J. Cheminform. 6:25. 10.1186/1758-2946-6-25 24910716 PMC4047268

[B113] Sahoo S. Aurich M. K. Jonsson J. J. Thiele I. (2014). Membrane transporters in a human genome-scale metabolic knowledgebase and their implications for disease. Front. Physiol. 5:91. 10.3389/fphys.2014.00091 24653705 PMC3949408

[B114] Salim N. Holliday J. Willett P. (2003). Combination of fingerprint-based similarity coefficients using data fusion. J. Chem. Inf. Comp. Sci. 43, 435–442. 10.1021/ci025596j 12653506

[B115] Saubern S. Guha R. Baell J. B. (2011). KNIME workflow to assess PAINS filters in SMARTS format. comparison of RDKit and indigo cheminformatics libraries. Mol. Inform. 30, 847–850. 10.1002/minf.201100076 27468104

[B116] Sedykh A. Fourches D. Duan J. M. Hucke O. Garneau M. Zhu H.. (2013). Human intestinal transporter database: QSAR modeling and virtual profiling of drug uptake, efflux and interactions. Pharm. Res. 30, 996–1007. 10.1007/s11095-012-0935-x 23269503 PMC3596480

[B117] Senger S. (2009). Using tversky similarity searches for core hopping: finding the needles in the haystack. J. Chem. Inf. Model. 49, 1514–1524. 10.1021/ci900092y 19453147

[B118] Sneath P. H. A. Sokal R. R. (1973). Numerical Taxonomy. San Francisco, CA: Freeman.

[B119] Sterling T. Irwin J. J. (2015). ZINC 15 - ligand discovery for everyone. J. Chem. Inf. Model. 55, 2324–2337. 10.1021/acs.jcim.5b00559 26479676 PMC4658288

[B120] Sugiyama Y. Steffansen B. (eds.). (2013). Transporters in Drug Development: Discovery, Optimization, Clinical Study and Regulation. New York, NY: AAPS/Springer.

[B121] Swainston N. Mendes P. Kell D. B. (2013). An analysis of a ‘community-driven’ reconstruction of the human metabolic network. Metabolomics 9, 757–764. 10.1007/s11306-013-0564-3 23888127 PMC3715687

[B122] Swamidass S. J. Baldi P. (2007). Bounds and algorithms for fast exact searches of chemical fingerprints in linear and sublinear time. J. Chem. Inf. Model. 47, 302–317. 10.1021/ci600358f 17326616 PMC2527184

[B123] Thiele I. Swainston N. Fleming R. M. T. Hoppe A. Sahoo S. Aurich M. K.. (2013). A community-driven global reconstruction of human metabolism. Nat. Biotechnol. 31, 419–425. 10.1038/nbt.2488 23455439 PMC3856361

[B124] Tomei S. Yuasa H. Inoue K. Watanabe J. (2001). Transport functions of riboflavin carriers in the rat small intestine and colon: site difference and effects of tricyclic-type drugs. Drug Deliv. 8, 119–124. 10.1080/107175401316906874 11570591

[B125] Tversky A. (1977). Features of Similarity. Psychol. Rev. 84, 327–352. 10.1037/0033-295X.84.4.327

[B126] Walters W. P. (2012). Going further than Lipinski's rule in drug design. Exp Opin. Drug Discov. 7, 99–107. 10.1517/17460441.2012.648612 22468912

[B127] Wang Y. A. Eckert H. Bajorath J. (2007). Apparent asymmetry in fingerprint similarity searching is a direct consequence of differences in bit densities and molecular size. Chem. Med. Chem. 2, 1037–1042. 10.1002/cmdc.200700050 17506042

[B128] Wang Y. Bajorath J. (2008). Balancing the influence of molecular complexity on fingerprint similarity searching. J. Chem. Inf. Model. 48, 75–84. 10.1021/ci700314x 18081268

[B129] Willett P. (2006). Similarity-based virtual screening using 2D fingerprints. Drug Discov. Today 11, 1046–1053. 10.1016/j.drudis.2006.10.005 17129822

[B130] Willett P. (2014). The calculation of molecular structural similarity: principles and practice. Mol. Inform. 33, 403–413. 10.1002/minf.201400024 27485978

[B131] Willett P. Barnard J. M. Downs G. M. (1998). Chemical similarity searching. J. Chem. Inf. Comp. Sci. 38, 983–996. 10.1021/ci9800211

[B132] Winter G. E. Radic B. Mayor-Ruiz C. Blomen V. A. Trefzer C. Kandasamy R. K.. (2014). The solute carrier SLC35F2 enables YM155-mediated DNA damage toxicity. Nat. Chem. Biol. 10, 768–773. 10.1038/nchembio.1590 25064833 PMC4913867

[B133] Wold S. Sjöström M. Eriksson L. (2001). PLS-regression: a basic tool of chemometrics. Chemometr. Intell. Lab. Syst. 58, 109–130. 10.1016/S0169-7439(01)00155-1

[B134] You G. Morris M. E. (eds.). (2014). Drug Transporters: Molecular Characterization and Role in Drug Disposition. New York, NY: Wiley.

[B135] Zhu X. Goldberg A. B. (2009). Introduction to Semi-Supervised Learning. San Rafael, CA: Morgan & Claypool.

